# From Metabolites to Mechanisms: *Scorzonera parviflora* Aerial Parts and Roots Extracts Profiled by UPLC‐ESI‐MS/MS, In Vitro/In Silico Tests, and Network Analysis

**DOI:** 10.1002/fsn3.72092

**Published:** 2026-07-09

**Authors:** Gokhan Zengin, Nilofar Nilofar, Gunes Ak, Eliana Fernandes, Luísa Custódio, Maria João Rodrigues, Shaza Aly, Omayma A. Eldahshan, Abdel Nasser Singab, Evren Yildiztugay, Milena Terzic, Meng‐Yao Li

**Affiliations:** ^1^ Department of Biology, Science Faculty Selcuk University Konya Turkey; ^2^ Department of Pharmacy, Botanic Garden “Giardino dei Semplici” “G. d'Annunzio” University Chieti Italy; ^3^ Centro de Ciências do Mar do Algarve (CCMAR/CIMAR LA), Campus de Gambelas Universidade do Algarve Faro Portugal; ^4^ Department of Pharmacognosy, Faculty of Pharmacy Badr University in Cairo Cairo Egypt; ^5^ Department of Pharmacognosy, Faculty of Pharmacy Ain Shams University Cairo Egypt; ^6^ Center of Drug Discovery Research and Development Ain Shams University Cairo Egypt; ^7^ Department of Biotechnology, Science Faculty Selcuk University Konya Turkey; ^8^ Faculty of Technology Novi Sad University of Novi Sad Novi Sad Serbia; ^9^ State Key Laboratory of Systems Medicine for Cancer, Shanghai Cancer Institute, Renji Hospital Shanghai Jiao Tong University School of Medicine Shanghai China; ^10^ Shanghai Key Laboratory for Cancer Systems Regulation and Clinical Translation Shanghai Jiading District Central Hospital Shanghai China

**Keywords:** antioxidant, cholinesterase, cytotoxicity, *Scorzonera parviflora*, tyrosinase, UPLC/MS

## Abstract

In the present study, the extraction of *Scorzonera parviflora* aerial parts and roots was performed using four solvents, namely ethyl acetate (EA), ethanol, ethanol/water, and water. The total phenolic and flavonoid contents were evaluated by Folin–Ciocalteu and AlCl₃ assays, while the chemical profile of extracts was determined by ultra performance liquid chromatography tandem mass spectrometry (UPLC–MS/MS). Antioxidant activities were assessed by radical scavenging, reducing power and metal chelating assays, whereas enzyme inhibition activity was tested against cholinesterases (acetylcholinesterase (AChE), butyrylcholinesterase (BChE)), tyrosinase, and carbohydrate‐digesting enzymes (*α*‐amylase and *α*‐glucosidase). Additionally, molecular docking and network pharmacology analyses were conducted to elucidate the potential targets and mechanisms underlying the observed bioactivities. The enzyme inhibition, cytotoxicity and network pharmacology components were combined within a single study to link the chemical profile of the extracts to their multi‐target biological effects and to identify the molecular targets underlying the observed activities. The results showed that the highest total phenolic content was found in the aerial part water extract (33.28 mg gallic acid equivalent (GAE)/g), followed by the ethanol/water extract (29.63 mg GAE/g), while the EA extract contained the highest amount of flavonoids (22.88 mg rutin equivalent (RE)/g). The phytochemical profile consisted of several classes, with flavonoids being the most abundant, followed by phenolic acids, glycosides and fatty acids. Aerial part extracts exhibited higher antioxidant activity than root extracts in most assays. Among all extracts, the aerial part water extract had the highest 2,2′‐azinobis(3‐ethylbenzothiazoline‐6‐sulfonic acid) (ABTS) (89.44 mg trolox equivalent (TE)/g) and metal chelating activity (MCA) (19.31 mg ethylenediaminetetraacetate equivalent (EDTAE)/g), while the ethanol/water extract demonstrated the strongest overall antioxidant capacity. The highest inhibition of AChE and BChE was observed for the roots ethanol extracts, measuring 3.04 mg and 3.66 mg galantamine equivalent (GALAE)/g, respectively. The ethanol/water extracts displayed the strongest tyrosinase inhibition, recorded with 59.57 mg kojic acid equivalent (KAE)/g. In conclusion, *S. parviflora* extracts exhibit notable antioxidant and enzyme inhibitory activities, highlighting their remarkable therapeutic potential, making them promising candidates for applications in the pharmaceutical, nutraceutical and natural product industries.

## Graphical abstract

*Scorzonera parviflora* is considered a promising candidate for applications in the pharmaceutical, nutraceutical, and natural product industries.

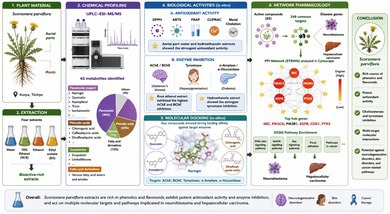

## 1 Introduction

Antioxidants and enzyme inhibitors represent two distinct classes of bioactive compounds that have attracted significant scientific interest over the past decade due to their demonstrated health‐promoting properties (Eldahshan et al. 2025; Aly et al. 2025; Wu, Chen, et al. 2025). Antioxidants function as defensive mechanisms against oxidative stress by neutralizing free radicals, thereby mitigating pathological processes associated with major diseases, including cancer, diabetes, and cardiovascular conditions (Mutlu et al. 2025; Wu, Liang, et al. 2025; Bai et al. 2025). Enzyme inhibitors modulate key enzymatic activities implicated in obesity, diabetes, dermatological disorders, and neurodegenerative diseases like Alzheimer's, consequently alleviating clinical manifestations (Acet 2021; Aly et al. 2022; Mei et al. 2024; Zong et al. 2025). Although numerous synthetic antioxidants and enzyme inhibitors have been developed, many exhibit adverse long‐term side effects (Nilofar, Bahadırlı, et al. 2024; Mostafa et al. 2016), creating an urgent demand for safer natural alternatives. Plants serve as abundant renewable sources of such bioactive compounds, with phenolics, flavonoids, alkaloids, and terpenoids demonstrating potent antioxidant capacities and enzyme‐inhibiting activities that hold promise for improving human health outcomes (Nilofar, Bahadırlı, et al. 2024; Mostafa et al. 2018; Aly et al. 2021; Mahomoodally et al. 2025).

The genus *Scorzonera* L. belongs to the tribe Cichorieae within Asteraceae family. The plants are mainly distributed in arid regions of Eurasia, Africa and southern and central Europe (Duran and Hamzaoglu 2004; Karaer and Celep 2007). Many species are endemic to Anatolia (Turkey) (Erik, Yaylı, et al. 2021; Acikara et al. 2014; Sarı et al. 2019), China (Li et al. 2004; Zhu et al. 2009) and Mongolia (Tsevegsuren et al. 2011; Wang et al. 2012). There are approximately 180–190 species in this genus. Phenolic acid derivatives (Granica and Zidorn 2015; Bader et al. 2011), flavonoids (Bader et al. 2011; Benabdelaziz et al. 2014; Xie et al. 2016), dihydroisocoumarins (Şahin et al. 2020; Sarı et al. 2007), triterpenoids (Çetin et al. 2018; Erik, Coşkunçelebi, et al. 2021), sesquiterpenoids (Zhu et al. 2010), and several other bioactive compounds have been reported from the genus. *S. parviflora* is widely distributed, especially in temperate areas of Afghanistan, Western Serbia and Europe (Dítě et al. 2015). Plants of this species also occur in central and eastern Anatolia. As with other members of the genus, *S. parviflora* is called “tekesakalı” and “yemlik” in Turkish. Most of the species of the genus *Scorzonera* are components of Turkish folk medicine and used for various diseases such as diabetes, hypertension, atherosclerosis, rheumatism, kidney diseases, atherosclerosis and pains. However, there is no specific folk medicinal use for *S. parviflora* (Karakaya et al. 2020; Baytop 1999; Yaldiz and Çalişkan 2017).

Regarding metabolomics studies and chemical characterization of *S. parviflora* , only a few research works have been reported despite its worldwide growth. The aqueous methanolic extract of *S. parviflora* was found to contain luteolin‐7‐*O*‐glucoside, hyperoside, dicaffeoylquinic acid, and chlorogenic acid by HPLC (Zidorn et al. 2003; Akkol et al. 2012). In fact, several common triterpenoids, including lupeol, lupeol acetate, and taraxasteryl acetate, were determined in n‐hexane extracts from the aerial parts and roots of the plant by HPLC (Bahadır‐Acıkara et al. 2018). Several studies have been conducted to understand the potential medicinal properties of *S. parviflora* . However, experiments testing for anti‐inflammatory and analgesic effects on mice did not produce significant results with the plant extract. Likewise, no evidence was found that the plant promotes wound healing. In other experiments, a methanol extract of *S. parviflora* showed antioxidant activity. An extract from the roots of the plant was more potent in this regard than an extract from its aerial parts (Acikara et al. 2013). A study investigating the hepatoprotective ability of the plant yielded promising results. Treating mice with chemically induced liver injury with *S. parviflora* appeared to help repair damaged cells (Özbek et al. 2017). This protective effect is thought to be related to the presence of chlorogenic acid.

More recently, Acıkara et al. (2025) reported a phytochemical study of *S. parviflora* roots collected in Gölbaşı (Ankara, Türkiye), in which bioactivity‐guided fractionation of the ethyl acetate fraction led to the isolation and full structural elucidation (by MS, IR and NMR) of three previously undescribed phenolic acid derivatives (parvifloric acids A–C) and a new sesquiterpene lactone (parviflorin), together with seven known compounds, with 3,5‐*O*‐dicaffeoylquinic acid being the most active in the ABTS and DPPH radical‐scavenging assays. That work was therefore directed at the isolation and structural characterization of novel constituents from the roots only, using antioxidant capacity as the sole biological endpoint. In contrast, the present study is complementary in scope: it provides a comparative UPLC‐ESI‐MS/MS metabolomic profile of both the aerial parts and the roots obtained with four solvents of differing polarity, and couples this profiling with a markedly broader bioactivity assessment—antioxidant, multi‐enzyme inhibitory and cytotoxic assays—together with molecular docking and network pharmacology, so as to relate the overall chemical composition of the extracts to their potential mechanisms of action. The two studies thus address different but mutually reinforcing questions for this species.

The present study was designed to investigate the chemical profiling of aerial and root extracts of *S. parviflora* from the Turkish flora using UPLC‐ESI/MS–MS. In addition, their antioxidant activities, enzyme inhibitory potential against a series of enzymes related to important human disorders, as well as cytotoxic effects on human embryonic kidney cells (HEK 293), human hepatocarcinoma cells (HepG2), and SHSY5Y cell lines were evaluated. The enzyme inhibition panel, the cytotoxicity panel, and the network pharmacology analysis were deliberately combined within a single study so that the work would move beyond descriptive screening: the in vitro enzyme‐inhibitory and cytotoxic assays define the functional profile of the extracts, while the network pharmacology and molecular docking link the metabolites identified by UPLC‐ESI‐MS/MS to candidate protein targets and pathways, thereby providing a mechanistic rationale for the observed antioxidant, enzyme‐inhibitory, and cytotoxic effects within one coherent dataset.

## 2 Materials and Methods

### 2.1 Plant Materials and Extraction

Plant materials of *S. parviflora* were gathered in 2023 from around Terkasan Lake (930 m, Cihanbeyli, Konya, Turkey). The taxonomic confirmation was performed by Dr. Evren Yildiztugay, and a voucher specimen was stored in the herbarium of Selcuk University (Voucher number: EY‐3436). The aerial parts and roots were separated, dried in the shade at room temperature, powdered, and then stored away from light.

Four solvent systems were used to obtain bioactive constituents: ethyl acetate, ethanol, 70% (*v/v*) ethanol–water, and distilled water. For each extraction, 10 g of the prepared plant material was mixed with 200 mL of the corresponding solvent. The methodology was adapted according to the properties of the solvent. Extractions using organic solvents were performed via maceration for 24 h under ambient conditions. In contrast, the water‐based extraction utilized a 15‐min infusion with heated water. Following extraction, the products obtained were concentrated using distinct techniques. The aqueous extract was stabilized by lyophilization, while the organic solvent fractions were recovered by evaporating the solvents under reduced pressure using a rotary evaporator.

### 2.2 Determination of Total Phenolic and Flavonoid Contents

Total phenolic and flavonoid contents of the extracts were determined using established colorimetric assays, following the referenced procedure. Calibration curves were constructed using gallic acid (mg gallic acid equivalents (GAE)/g) and rutin (mg rutin equivalents (RE)/g) as standards (Bibi Sadeer et al. 2020).

### 2.3 Phytochemical Analysis by UPLC‐ESI‐MS/MS Analysis

The phytochemical profile of *S. parviflora* aerial parts and roots extracts was analyzed using high‐performance liquid chromatography coupled with ESI‐MS/MS detection, following a previously reported method (Aly, Elissawy, Salah, et al. 2023; Aly, Elissawy, et al. 2024; Aly, Elissawy, Mahmoud, et al. 2023). The details are given in the supplemental materials.

### 2.4 Determination of In Vitro Antioxidant Properties

Antioxidant capacity was assessed using a panel of in vitro assays, following a previously described protocol (Grochowski et al. 2017). Reducing power was evaluated using FRAP (ferric reducing antioxidant power) and CUPRAC (cupric reducing antioxidant capacity), while radical scavenging was measured using DPPH (2,2‐diphenyl‐1‐picrylhydrazyl) and ABTS (2,2′‐azinobis(3‐ethylbenzothiazoline‐6‐sulfonic acid)) assays. Results from these four assays were quantified and standardized to Trolox and reported as mg Trolox equivalents (TE) per g of dried extract (mg TE/g). Trolox was expressed as milligrams of Trolox equivalent per gram of dried extract (mg TE/g). Total antioxidant capacity was additionally determined by the phosphomolybdenum method (PBD assay) and expressed as mmol Trolox equivalents per g (mmol TE/g). Finally, metal‐chelating activity was evaluated using a chelation assay and reported as mg EDTA equivalents (EDTAE) per g of extract (mg EDTAE/g). The experimental details are provided in Supporting Information.

### 2.5 Determination of Inhibitory Effects Against Some Key Enzymes

The enzyme inhibitory potential of the extracts was evaluated against five key targets: acetylcholinesterase (AChE), butyrylcholinesterase (BChE), tyrosinase, *α*‐amylase and *α*‐glucosidase, using established colorimetric procedures (Grochowski et al. 2017). To standardize the results, inhibition was quantified using established reference compounds. Cholinesterase inhibitory activity was expressed as mg galanthamine equivalents (GALAE) per g of extract (mg GALAE/g). *α*‐Amylase and *α*‐glucosidase inhibitory activities were expressed as mg acarbose equivalents (ACAE) per g (mg ACAE/g), while tyrosinase inhibition was reported as mg kojic acid equivalents (KAE) per g (mg KAE/g). The experimental details are provided in Supporting Information.

### 2.6 Determination of Cytotoxic Potential

The human cell lines HepG2 (hepatocellular carcinoma), SH‐SY5Y (neuroblastoma), and HEK 293 (embryonic kidney) were cultured in the laboratory under standard conditions, following the procedure described by Rodrigues et al. (2016). To assess how toxic the plant extracts were to cells, we first seeded them in 96‐well plates (5000 cells per well) and let them settle overnight. The next day, we treated the cells with the extracts (100 μg/mL) for 72 h. After this treatment, we added a yellow MTT dye to the wells. Living cells convert this dye into a purple formazan product. Two hours later, we dissolved the purple crystals and measured the resulting color intensity with a plate reader. The darker the purple, the more living cells were present. We then compared these results to untreated control cells to calculate the percentage of cells that survived the extract treatment.

### 2.7 Molecular Docking

To identify the biologically active compounds of *S. parviflora* , a computational workflow was established. The initial geometry optimization and energy minimization of all candidate molecules were performed using Chem3D v22.2.0 with the MM2 force field to obtain stable low‐energy conformations. The three‐dimensional structures of related target proteins were obtained from the Protein Data Bank (https://www.rcsb.org/). These protein models were prepared in PyMOL v3.1.6.1 by removing crystal water and bound native ligands and adding polar hydrogen atoms to define correct ionization states. Subsequently, molecular docking simulations were carried out using AutoDock Vina 1.20 to predict binding affinity and interaction patterns. The output poses were analyzed and visualized in Maestro View v14.5, and two‐dimensional interaction diagrams were generated for further interpretation of key binding contacts.

### 2.8 Network Pharmacology

The phytochemical composition of *S. parviflora* was systematically profiled using UPLC–MS/MS analysis. On the basis of the detected components, some compounds were chosen for the subsequent prediction of potential gene targets. Following compound identification, the corresponding chemical structures were retrieved from PubChem (https://pubchem.ncbi.nlm.nih.gov/) to obtain their Canonical Identifier (CID) numbers. These CIDs, representing the specific chemical structures, were then submitted to Swiss Target Prediction (http://swisstargetprediction.ch/) for target prediction. Predicted targets with a probability score of zero were removed from the dataset. The remaining targets for each compound were consolidated into a single dataset and visualized as an interaction network using Cytoscape 3.10.2. In this network, node color indicates target classification, while node size reflects the degree value, which ranges from 60 to 150.

In order to screen for potential target genes related to cervical adenocarcinoma and colorectal adenocarcinoma, a systematic multi database mining method was used. GeneCards (https://www.genecards.org/), the Therapeutic Target Database (TTD, https://db.idrblab.net/ttd), DrugBank (https://go.drugbank.com/), the Comparative Toxicogenomics Database (CTD, https://ctdbase.org/) and Online Mendelian Inheritance in Man (OMIM, https://omim.org) were searched Using “Neuroblastoma” and “Hepatocellular carcinoma” as keywords to obtain gene targets. The obtained Target entries from DrugBank were cross‐checked with UniProt (https://www.uniprot.org/) to confirm human protein IDs and biological relevance. All retrieved targets were merged and duplicates removed to generate a non‐redundant set of genes. The compound gene associations and tumor‐specific neuroblastoma and hepatocellular carcinoma gene expression profiles were integrated and visualized using the “Wei Sheng Xin” online platform (https://www.bioinformatics.com.cn/). A three‐set Venn diagram was constructed to show overlapping genes among candidate compounds, neuroblastoma and hepatocellular carcinoma, highlighting common therapeutic targets.

To characterize potential direct and indirect interactions between the identified target proteins, a protein–protein interaction (PPI) network was constructed using the STRING database (https://cn.string‐db.org/). The analysis was restricted to *Homo sapiens* and intersecting genes from the compound, neuroblastoma, and hepatocellular carcinoma datasets. A confidence threshold of 0.900 was applied to retain only high‐confidence interactions for network assembly, after which isolated nodes with no connections were excluded. The resulting interaction data were imported into Cytoscape 3.10.2. The “Analyze Network” function was used to calculate the degree centrality of each node. For visualization purposes, node size was scaled proportionally to its degree value, with diameters ranging from 60 to 150 pixels. Node color was also assigned using a continuous gradient based on degree, allowing for intuitive interpretation of each node's relative influence and centrality within the network.

To investigate the enrichment relationship between *S. parviflora* and the two tumor types, GO enrichment analysis was conducted using Metascape (http://metascape.org/gp/index.html). The analysis covered all three major Gene Ontology categories: biological process (BP), molecular function (MF), and cellular component (CC). After obtaining the GO output, the log10‐transformed *p* values were converted back to their original values, and only terms with *p* ≤ 0.05 were retained. The enrichment scores were then ranked by statistical significance, and the top 10 pathways in each category were selected, collated, and summarized. Finally, the enrichment outcomes were visualized using the Wei‐Sheng‐Xin online platform (https://www.bioinformatics.com.cn/).

For Kyoto Encyclopedia of Genes and Genomes (KEGG) pathway analysis, the latest KEGG pathway gene annotations were retrieved via the KEGG API (https://www.kegg.jp/kegg/rest/) and used as the reference background for mapping gene sets and subsequent interpretation. Functional enrichment of the target gene sets was conducted with the “clusterProfiler v3.14.3” package in R, restricting gene set sizes to between 5 and 5000 genes. Pathways were deemed significantly enriched when they met the dual criteria of *p* < 0.05 and a false discovery rate (FDR) < 0.25.

### 2.9 Statistical Analysis

To ensure reliability, every test was performed in three separate trials. The results are presented as the mean value alongside the standard deviation. We analyzed this data for statistical significance using ANOVA followed by a Tukey test, with a *p*‐value of less than 0.05 considered significant. All statistical work was done with GraphPad Prism, version 9.2.

## 3 Results and Discussion

### 3.1 Total Phenolic and Flavonoid Content

Phenolic compounds are phytochemicals widely distributed in vegetables, fruits, and herbs, being responsible for the color, flavor, and quality of these foods. The differences found in phenolic content between leaves and roots can be attributed to different extraction processes, analysis methods, or even environmental influences such as temperature, drought, light, pollution, and pathogens (Ferreyra et al. 2023). The extraction of these compounds from the plant matrix is highly dependent on the solvent used (Do et al. 2014).

In the present study, the total phenolic content and total flavonoid content of different extracts from aerial parts and roots of *S. parviflora* showed significant variation (Table 1). Among tested extracts, the water extract exhibited the highest TPC value at 33.28 mg GAE/g followed by ethanol/water with 29.63 mg GAE/g, while EA and ethanol extracts revealed relatively lower values. On the other hand, results for TFC indicated that the EA extract had the highest concentration of flavonoids at 22.88 mg RE/g. These results are in agreement with previous studies (Ak et al. 2020) which also documented notably high TFC levels in EA extract (17.49 mg RE/g) compared to other extracts of *S. hispanica* . Similarly, another study (Ahmed et al. 2025) also demonstrated that the EA extract has a higher ability to extract flavonoid compounds than other solvent fractions, further supporting their efficiency in recovering phenolic constituents. The ethanol and ethanol/water extracts showed similar TFC values, around 16 mg RE/g for the latter, with a minimum recorded for the water extract at 15.54 mg RE/g.

**TABLE 1:** Extraction yields, total phenolic and flavonoid content in the tested extracts of *S. parviflora* .

Parts	Extracts	Extraction yields (%)	TPC (mg GAE/g)	TFC (mg RE/g)
Aerial parts	Ethyl acetate	3.04	17.35 ± 1.15^e^	22.88 ± 0.48^a^
	Ethanol	4.64	22.02 ± 0.07^c^	16.36 ± 0.21^c^
	Ethanol/Water	20.04	29.63 ± 0.43^b^	16.93 ± 0.07^b^
	Water	11.92	33.28 ± 0.66^a^	15.54 ± 0.06^d^
Roots	Ethyl acetate	2.41	19.75 ± 0.12^d^	0.88 ± 0.04^e^
	Ethanol	5.16	16.90 ± 0.07^e^	1.08 ± 0.03^e^
	Ethanol/Water	22.32	19.21 ± 0.24^d^	1.22 ± 0.05^e^
	Water	10.97	16.08 ± 0.14^e^	1.01 ± 0.01^e^

*Note:* Values are reported as mean ± SD of three parallel measurements. For each assay, values followed by different letters (a–e) are significantly different at *p <* 0.05 (Tukey's test).

Abbreviations: GAE, gallic acid equivalent; RE, rutin equivalent.

For root extracts, TPC values were relatively comparable but lower than those observed in aerial parts. The highest values were recorded in EA and ethanol/water extracts, yielding 19.75 mg GAE/g and 19.21 mg GAE/g, respectively. However, the TFC content of roots was significantly lower. Similarly, a previous study reported that *S. hispanica* root extracts using EA solvent exhibited the highest TPC levels (Ak et al. 2020). Our findings corroborate this trend, demonstrating higher phenolic concentrations in aerial parts compared to roots, which aligns with previous observations (Dall'Acqua, Ak, Sut, Ferrarese, et al. 2020). These results further confirm established patterns indicating greater phenolic accumulation in aerial tissues relative to root tissues (Zengin, Leyva‐Jiménez, et al. 2024; Emir et al. 2025).

### 3.2 UPLC/MS Analysis of Different Extracts of *S. parviflora* Aerial Parts and Roots

The UPLC‐ESI/MS–MS analysis of ethyl acetate, methanol, ethanol/aqueous, and aqueous extracts from the aerial parts and roots of *S. parviflora* generated comprehensive phytochemical profiles in both negative and positive ion modes (Figures S1–S8), enabling the tentative identification of 65 secondary metabolites (Table 2). This extensive characterization revealed a diverse array of compound classes including phenolic acids, flavonoids, fatty acids and their derivatives, coumarins, and various other metabolites, thereby underscoring the species' significant chemotaxonomic value and potential therapeutic applications.

**TABLE 2:** The HPLC‐ESI/MS–MS‐based characterization of phytoconstituents of the various extracts of *S. parviflora* aerial parts and roots in both negative and positive ionization modes.

Peak no.	*t*_R_	[M‐H]^−^	[M + H]^+^	MS^2^	Tentatively identified compounds	Phytochemical class	Aerial parts				Roots				References
							EtOAc	EtOH	EtOH/W.	Water	EtOAc	Ethanol	EtOH/W.	Water	
1	0.66	341	−	244, 195, 179	Sucrose^a^	Sugar	−	+	−	−	+	+	−	−	Dall'Acqua, Ak, Sut, et al. (2020)
2	0.70	377	−	353, 191, 179, 161, 119, 341	Quinic acid derivative	Phenolic acid	−	+	+	+	−	+	−	+	Cusumano et al. (2024)
3	0.71	271	−	191, 176, 104	Naringenin	Flavonoid	−	−	−	−	−	+	−	−	Zengin, Yagi, et al. (2024)
4	0.97	−	116	105	Proline	Amino acid	−	−	+	−	−	−	−	−	Mohsen et al. (2020)
5	1.04	191	−	253, 173, 162, 153, 130	Quinic acid	Organic acid	−	−	+	+	−	−	−	+	Goher et al. (2024)
6	1.65	−	188	170, 146, 118	Tryptophan derivative	Amino acid	−	−	+	−	−	−	−	−	Khattab et al. (2023)
7	2.10	353	−	191	Chlorogenic acid^a^	Phenolic acid	−	−	+	−	−	−	−	−	Zidorn et al. (2003)
8	2.44	707	−	353, 191	Caffeoylquinic acid dimer^a^	Phenolic acid	−	−	+	−	−	−	−	−	Dall'Acqua, Ak, Sut, et al. (2020)
9	2.46	−	163	145, 135, 117	Umbelliferone^a^	Coumarin	−	−	+	+	−	−	−	+	Wu et al. (2018)
10	2.81	353	−	191	Caffeoylquinic acid^a^	Phenolic acid	−	+	+	+	−	+	−	+	Dall'Acqua, Ak, Sut, et al. (2020)
11	5.19	271	−	191, 176, 111	Naringenin isomer	Flavonoid	−	+	+	+	−	+	−	+	Zengin, Yagi, et al. (2024)
12	5.73	515	−	353, 191	Dicaffeoylquinic acid^a^	Phenolic acid	−	−	−	−	+	−	−		Granica et al. (2015)
13	5.76	−	193	178, 133, 122	Scopoletin^a^	Coumarin	−	−	−	−	−	−	+	+	Wu et al. (2018)
14	6.20	−	303	303, 285, 257, 229, 153, 137	Quercetin^a^	Flavonoid	−	−	+	−	−	−	−	−	Dall'Acqua, Ak, Sut, et al. (2020)
15	6.35	579	−	271, 151, 119	Naringin	Flavonoid	−	−	−	+	−	−	−	−	Nilofar, Zengin, et al. (2024)
16	6.42	447	−	239, 327, 284	Luteolin‐*C*‐hexoside^a^	Flavonoid	−	+	+	+	−	−	−	−	Granica et al. (2015)
17	6.58	341	−	341, 179, 133	Caffeic acid hexoside	Phenolic acid	−	−	−	−	−	+	−	−	Kramberger et al. (2020)
18	6.77	515	−	353, 191, 179	Dicaffeoylquinic acid isomer^a^	Phenolic acid	−	+	+	+	−	−	−	−	Granica and Zidorn (2015), Sarı (2010)
19	6.92	515	−	353, 179, 85	Dicaffeoylquinic acid isomer^a^	Phenolic acid	−	+	−	−	−	+	−	−	Granica and Zidorn (2015), Dall'Acqua, Ak, Sut, et al. (2020), Sarı (2010)
20	7.31	491	−	191, 167, 123	Vanillic Acid‐*O*‐dihexoside	Phenolic acid	−	−	−	−	−	+	−	−	Khattab et al. (2023)
21	7.35	255	−	193, 74, 59	Scorzoveratrin^a^	3‐Benzylphthalide	+	−	−	−	+	−	−		Sarı (2010)
22	7.54	303	−	300, 121	Dihydroquercitin	Flavonoid	−	−	−	+	−	−	−	−	Khattab et al. (2023)
23	7.69	455	−	453, 441, 359, 341, 337, 329, 299, 293, 188, 179	Oleanolic acid^a^	Fatty acid	−	−	+	−	−	−	−	−	Wu et al. (2018)
24	7.72	583	−	431, 285, 228 345, 216, 179, 87	Kaempferol‐*O*‐galloylrhamnoside	Flavonoid	−	+	+	−	−	+	−	−	Mohsen et al. (2020)
25	8.82	327	−	291, 239, 229, 211, 193, 183, 171, 127	Hydroxy‐trimethoxy flavone	Flavonoid	+	−	+	+	−	−	−	−	Nilofar, Zengin, et al. (2024)
26	8.85	−	235	217, 199, 189, 161	Oxo‐bisabola‐(2,10*E*)‐diene‐12‐al (puliglutone)^a^	Bisabolene sesquiterpene	+	+	+	+	+	−	−	−	Granica et al. (2015)
27	8.87	329	−	247, 167, 151, 103	Tricin^a^	Flavonoid	−	−	−	−	+	−	−	+	Wu et al. (2018)
28	9.21	285	−	273, 257, 217, 211, 199, 164, 151	Kaempferol^a^	Flavonoid	+	−	−	−	−	−	−	−	Dall'Acqua, Ak, Sut, et al. (2020)
29	9.36	329	−	229, 211, 171, 183, 99, 245, 157	Trihydroxy‐ octadecenoic acid	Fatty acid	+	+	+	+	+	+	−	−	AbouZeid et al. (2022)
30	10.30	315	−	300, 272, 269, 252, 151	Isorhamnetin	Flavonoid	+	−	−	−	+	−	−	−	Abdl Aziz et al. (2024)
31	10.67	327	−	301, 233, 284, 174, 129	Dihydroxy‐oxo‐12‐ octadecenoic acid	Fatty acid	−	−	−	−	+	−	−	−	Farag et al. (2013)
32	10.71	−	293	145, 107 275, 223, 95	Licanic acid	Fatty acid	−	−	−	−	+	−	−	−	Khattab et al. (2023)
33	11.41	309	−	271, 183, 115, 90	Eicosaenoic acid	Fatty acid	−	+	−	−	−	−	−	−	Zengin et al. (2022)
34	11.52	−	274	106, 256, 274	Hexadecasphinganine	Sphingolipid	−	+	+	−	−	−	−	−	Cusumano et al. (2024)
35	11.88	311	−	257, 175, 155	Dihydroxy‐linoleic acid	Fatty acid	−	+	−	−	+	−	−		Farag, Otify, et al. (2016)
36	12.01	−	247	187, 131, 118, 95, 81, 71	Hydroxydodecanedioic acid	Fatty acid	−	−	−	−	+	−	−	−	Fayek et al. (2025)
37	12.19	311	−	255, 145, 121, 109	Octadecenedioic acid	Fatty acid	−	+	−	−	−	−	−	−	Fayek et al. (2025)
38	13.54	−	302	284, 266, 254	Sphinganine	Sphingolipid	−	−	−	+	−	−	−	−	Khattab et al. (2023)
39	13.60	311	−	293, 276, 183, 162, 127	Dihydroxy‐linoleic acid	Fatty acid	+	−	−	−	−	−	−	−	Farag, Otify, et al. (2016)
40	14.35	293	−	249, 211, 183, 171, 121, 109	Oxo‐octadecadienoic acid^a^	Fatty acid	+	−	+	+	−	−	−	−	Granica et al. (2015)
41	14.77	−	280	263, 165, 119, 107, 109	Octadecadienoic acid	Fatty acid	+	+	+	−	+	−	+	−	AbouZeid et al. (2022)
42	14.86	291	−	195, 123	Methyl octadecadiynoate	Fatty acid	+	+	−	−	−	−	−	−	Fayek et al. (2025)
43	15.36	295	297	280,195, 177, 153,141, 121, 109	Hydroxy octadecadienoic acid (Hydroxy‐linoleic acid)	Fatty acid	+	+	+	+	+	+	+	−	Zengin, Yagi, et al. (2024)
44	15.92	311	−	295, 261, 217, 128	Octadecenedioic acid	Fatty acid	+	−	−	−	−	−	−	−	Farag, El Fishawy, et al. (2016)
45	15.95	293	295	181, 149, 135, 121, 109, 107	13‐Hydroxy‐octadecatrienoic acid^a^	Fatty acid	+	−	+	−	+	+	−	−	Granica et al. (2015)
46	16.33	293	−	275, 249, 197, 185, 125	13‐Oxo‐(9*E*,11*E*)‐octadecadienoic acid^a^	Fatty acid	+	−	+	−	−	−	−	−	Granica et al. (2015)
47	16.55	−	280	263, 165, 119, 107	Octadecadienoic acid amide	Fatty acid amide	+	+	−	−	−	−	−	−	Farag, Otify, et al. (2016)
48	16.83	297	299	279, 274, 253, 171	Hydroxy octadecenoic acid (Hydroxy‐oleic acid)	Fatty acid	+	+	−	−	+	−	−	−	Ayoub et al. (2021)
49	17.11	295	−	277, 183, 171	Hydroxy octadecadienoic acid isomer	Fatty acid	+	+	−	−	−	−	−	−	Zengin, Yagi, et al. (2024)
50	17.40	−	228	43, 57, 74, 88, 102, 144, 187	Myristamide	Fatty acid amide	+	+	−	−	+	−	−	−	Zengin, Yagi, et al. (2024)
51	17.44	313	−	255, 225, 155	Dihydroxydimethoxyflavone	Flavonoid	+	−	−	−	+	+	−	−	Zengin et al. (2022)
52	18.76	579	−	287, 449 [M + H‐132‐162]	Scorzopygmaecoside^a^	Dihydroisocoumarin	−	−	−	−	+	−	−	−	Şahin et al. (2020)
53	18.82	−	280	263, 165, 119, 107	Octadecadienoic acid amide	Fatty acid amide	+	−	+	+	+	−	+	+	Farag, Otify, et al. (2016)
54	20.47	−	256	163, 102, 144, 130	Palmitamide	Fatty acid amide	+	+	+	+	+	−	+	+	Zengin, Yagi, et al. (2024)
55	20.68	623	−	321, 315, 300	Isorhamnetin‐*O*‐hexosyl‐*O*‐deoxyhexoside	Flavonoid	−	+	−	−	−	−	−	−	Zengin, Yagi, et al. (2024)
56	20.95	−	282	240, 121, 114, 111	Oleamide	Fatty acid amide	+	+	+	+	+	−	+	+	Zengin, Yagi, et al. (2024)
57	21.24	313	−	283, 183	Dihydroxy‐octadecenoic acid	Fatty acid	+	−	−	−	−	−	−	−	Farag et al. (2014)
58	22.29	−	441	384, 315, 285, 271,257, 229, 153, 137	24‐Ethyl‐Cholesta‐4, 9(11), 24(28)‐triene‐diol‐3‐one	Steroid	−	+	−	−	−	−	−	−	Farag et al. (2024)
59	22.84	−	481 [M + Na]^+^	325	(6‐*Trans*‐*p*‐coumaroyl)‐3‐*O*‐*β*‐D‐glucopyranosyl‐2‐deoxy‐D‐riburonic acid^a^	Phenolic acid	−	−	−	−	+	−	−	−	Milella et al. (2014)
60	22.96	327	−	282, 281, 270, 198	Trihydroxy octadecadienoic acid	Fatty acid	+	+	−	−	−	−	−	−	Farag, Otify, et al. (2016)
61	23.68	−	284	242, 163, 116, 114, 111, 102	Stearamide	Fatty acid amide	+	+	+	+	+	−	+	+	Zengin, Yagi, et al. (2024)
62	23.95	471	−	409, 393	Hydroxy‐betulinic acid^a^	Triterpenoid	−	−	−	−	+	−	−	−	Dall'Acqua, Ak, Sut, et al. (2020)
63	25.25	459	−	−	Tyrolobibenzyl B^a^	Tyrolobibenzyl	−	+	−	−	−	−	−	−	Zidorn et al. (2003)
64	25.65	−	439	393, 203	Olean‐ene‐dione	Triterpenoid	−	−	−	−	+	−	−	−	Fayek et al. (2025)
65	25.96	461	−	313, 299, 284, 256, 227, 188	Disometin‐*O*‐hexoside^a^	Flavonoid	−	+	−	−	−	+	−	−	Dall'Acqua, Ak, Sut, et al. (2020)

^a^ Previously identified compounds in the genus *Scorzonera*.

#### 3.2.1 Flavonoids

The LC/MS chromatograms of the two *S. parviflora* parts were dominated by flavonoids and their glycosides, including a series of flavanones such as naringenin at *m/z* 271 [M‐H]^−^ and its corresponding glycoside naringin at *m/z* 579 [M‐H]^−^ with fragment *m/z* 271 attributed to the loss of hexose unit [M‐162]^−^ and deoxyhexose unit [M‐146]^−^, flavonols like quercetin, kaempferol and isorhamnetin with *m/z* 303 [M + H]^+^, 285 [M‐H]^−^ and 315 [M‐H]^−^, respectively. Along with flavones luteolin‐*C*‐hexoside at *m/z* 447 [M‐H]^−^ that showed a neutral loss of 120 and 90 amu, indicative of a mono *C*‐ hexoside and tricin at *m/z* 329 [M‐H]^−^. The occurrence of different glycosylated forms, for example, isorhamnetin‐*O*‐hexosyl‐*O*‐deoxyhexoside at *m/z* 623 [M‐H]^−^ with fragment *m/z* 315 attributed to the loss of characteristic ions [M‐162]^−^ and [M‐146]^−^, kaempferol‐*O*‐galloylrhamnoside at *m/z* 583 [M‐H]^−^ that showed fragment ion at *m/z* 285 due to the loss of rhamnose and galloyl moieties [M‐146‐152]^−^, and diosmetin‐*O*‐hexoside at *m/z* 461 [M‐H]^−^ with fragment *m/z* 299 due to the loss of hexose moiety, underscored the complexity of its flavonoid metabolism. Flavonoid distribution showed distinct patterns across the different plant parts. The presence of tricin in roots and kaempferol, quercetin, naringin, luteolin‐*C*‐hexoside and kaempferol in aerial parts may contribute to the differential biological activities of extracts from these organs. Notably, kaempferol, quercetin and diosmetin‐*O*‐hexoside have been previously reported in an extract of *S. hieraciifolia* (Dall'Acqua, Ak, Sut, et al. 2020). In addition, tricin and luteolin‐*C*‐hexoside were identified in extracts of *S. divaricate* and *S. hispanica* , respectively (Wu et al. 2018; Granica et al. 2015). MS/MS spectra were crucial for tentative identification, as flavonoids typically exhibit fragmentation patterns characterized by neutral loss of glycosidic units (−162 Da for hexose, −146 Da for deoxyhexose) followed by retro Diels‐Alder (RDA) cleavage of the C‐ring, yielding diagnostic aglycone fragments. The mentioned neutral losses are typical of *O*‐glycosides, whereas *C*‐glycosides are distinguished by losses of 60, 90, 120, 150, and 180 Da (Aly, Elissawy, et al. 2024; Zengin, Yagi, et al. 2024; Aly, Mahmoud, et al. 2024).

#### 3.2.2 Phenolic Acids

This study revealed a wide and rich profile of phenolic acids, especially caffeoylquinic acid derivatives. chlorogenic acid *m/z* 353 [M‐H]^−^ with a characteristic fragment ion peak at *m/z* 191 corresponding to [C_7_H_11_O_6_]^−^ residue, caffeoylquinic acid *m/z* 353 [M‐H]^−^ showed the fragment peak at *m/z* 191 ([quinic acid‐H]^−^) due to the loss of a caffeoyl moiety.

Moreover, the dicaffeoylquinic acid at *m/z* 515 [M‐H]^−^ with a fragment ion at [M‐H‐caffeoyl]^−^ ion at *m*/*z* 353 through the neutral loss of 162 (C_9_H_6_O_3_). These phenolic acids were identified in different extracts, as previously reported for different *Scorzonera* species (Zidorn et al. 2003; Granica et al. 2015; Sarı 2010). These compounds are known to possess strong antioxidant properties and are considered chemotaxonomic markers for the *Asteraceae* family (Dall'Acqua, Ak, Sut, et al. 2020; Granica et al. 2015). Their prevalence, particularly in ethanol and hydroalcoholic extracts from both aerial parts and roots, suggests that *S. parviflora* is a valuable source of these health‐promoting phenolics. The identification of these compounds was confirmed by their characteristic MS^2^ fragmentation pattern, which generally produced a base peak at *m/z* 191 corresponding to the quinic acid moiety and a fragment at *m/z* 179 indicating the presence of the caffeic acid unit (Dall'Acqua, Ak, Sut, et al. 2020; Sarı 2010).

#### 3.2.3 Fatty Acids and Their Derivatives

A large proportion of the identified metabolites were fatty acids and derivatives, including hydroxy‐, oxo‐, and diunsaturated fatty acids, as well as fatty acid amides such as octadecadienamide, myristamide, palmitamide, oleamide, and stearamide. The high abundance of these lipophilic compounds, especially in EtOAc extracts, is consistent with their expected solubility and a common feature of plant metabolome profiles (Zengin, Yagi, et al. 2024; Cusumano et al. 2024). The identification of fatty acid amides such as oleamide, stearamide, and palmitamide is also noteworthy because this class of compounds has been linked to various biological effects (Zengin, Yagi, et al. 2024; Cusumano et al. 2024).

Furthermore, the analysis confirmed the presence of several compounds previously reported in the genus *Scorzonera*, serving as a validation of our methodology and reinforcing the chemotaxonomic links within the genus. These include bisabolene sesquiterpene (puliglutone), 3‐benzylphthalide (scorzoveratrin), dihydroisocoumarin (scorzopygmaecoside), and tyrolobibenzyl B. The detection of scorzoveratrin specifically in both EtOAc extracts from aerial parts and roots suggests it may be an important characteristic metabolite for *S. parviflora* . coumarins umbelliferone and scopoletin were also identified and have been previously characterized in *S. divaricate* (Wu et al. 2018).

The distribution of compounds across the extracts clearly demonstrates the effect of solvent polarity on metabolome coverage. The ethyl acetate and ethanol extracts contained a broader spectrum of medium‐to‐low polarity secondary metabolites, including flavonoids, fatty acids, and terpenoids. In contrast, the water and ethanol/water extracts contained higher concentrations of polar compounds, including specific phenolic acids such as quinic acid derivatives, chlorogenic acid, and caffeoylquinic acid, as well as amino acids such as proline and tryptophan derivatives. extraction efficiency depending on the solvent is important for guiding future bioactivity‐guided fractionation studies.

In conclusion, this UPLC‐ESI/MS–MS investigation presents the first comprehensive chemical characterization of *S. parviflora* , revealing a diverse array of bioactive compounds. The successful identification of multiple compounds, confirmed through their MS/MS fragmentation patterns, provides a solid foundation for elucidating the plant's potential therapeutic applications. The distinct chemical compositions observed between aerial and root components, coupled with the presence of genus‐specific markers, offer valuable insights for quality control and standardization of *S. parviflora* ‐derived products.

### 3.3 Determination of Antioxidant Properties

The antioxidant activities of various extracts from *S. parviflora* aerial parts and roots, as assayed by DPPH, ABTS, CUPRAC, FRAP, MCA, and PBD are presented in Table 3. The antioxidant capacity varied considerably and generally exhibited trends similar to those described for their TPC and TFC contents. Comparatively, extracts from the aerial parts demonstrated higher antioxidant activity than those from the roots across most assays. Among the aerial part extracts, the ethanol/water extract exhibited the highest antioxidant capacity in most assays, with values of 165.30 mg TE/g (CUPRAC), 89.97 mg TE/g (FRAP), and 52.81 mg TE/g (DPPH). The water extract showed comparable results, yielding values of 148.24 mg TE/g, 86.29 mg TE/g, and 51.45 mg TE/g, respectively. These findings align with previous studies (Do et al. 2014), which reported that both aqueous and organic solvent extractions yield strong antioxidant activity, likely due to their higher efficiency in extracting phenolic compounds. Caffeoylquinic acid derivatives identified by LC–MS in the ethanol/water extract (Table 2) are recognized for their potent antioxidant properties, as previously reported in different *Scorzonera* species (Do et al. 2014; Grishchenko et al. 2022; Lendzion et al. 2021). Dicaffeoylquinic acid, abundantly detected in the aerial parts of *S. parviflora* in this study, was previously reported by Acıkara et al. (2025) to exhibit significant antioxidant properties when extracted from the same plant species.

**TABLE 3:** Antioxidant properties of the tested extracts of *S. parviflora* .

Parts	Extracts	DPPH (mg TE/g)	ABTS (mg TE/g)	CUPRAC (mg TE/g)	FRAP (mg TE/g)	MCA (mg EDTAE/g)	PBD (mmol TE/g)
Aerial parts	Ethyl acetate	24.32 ± 1.08^g^	30.40 ± 1.34^e^	74.43 ± 2.31^e^	33.81 ± 1.07^g^	16.33 ± 0.05^c^	2.06 ± 0.06^ab^
	Ethanol	36.56 ± 1.11^d^	45.71 ± 0.51^c^	108.89 ± 2.79^c^	52.23 ± 0.36^d^	15.24 ± 0.37^d^	2.13 ± 0.10^a^
	Ethanol/Water	52.81 ± 0.03^a^	86.44 ± 0.31^a^	165.30 ± 1.20^a^	89.97 ± 0.81^a^	16.24 ± 0.09^c^	1.69 ± 0.10^c^
	Water	51.45 ± 0.20^ab^	89.16 ± 2.63^a^	148.24 ± 0.97^b^	86.29 ± 1.17^b^	19.13 ± 0.19^a^	1.72 ± 0.01^c^
Roots	Ethyl acetate	27.26 ± 1.32^f^	35.11 ± 0.95^d^	67.18 ± 1.19^f^	29.71 ± 0.28^h^	13.62 ± 0.11^e^	2.07 ± 0.08^ab^
	Ethanol	32.60 ± 0.64^e^	45.62 ± 0.78^c^	73.05 ± 0.15^e^	38.88 ± 0.19^f^	15.20 ± 0.24^d^	1.96 ± 0.06^ab^
	Ethanol/Water	49.84 ± 0.66^b^	69.27 ± 0.27^b^	95.90 ± 0.92^d^	56.02 ± 0.14^c^	16.17 ± 0.28^c^	1.88 ± 0.04^bc^
	Water	42.02 ± 1.19^c^	66.38 ± 0.22^b^	67.76 ± 0.65^f^	42.23 ± 0.45^e^	18.05 ± 0.07^b^	1.92 ± 0.02^b^

*Note:* Values are reported as mean ± SD of three parallel measurements. For each method, values followed by different letters (a–h) show significant differences at *p <* 0.05 (Tukey's test).

Abbreviations: EDTAE, EDTA equivalent; MCA, Metal chelating activity; PBD, Phosphomolybdenum; TE, Trolox equivalent.

The EA and ethanol extracts of the aerial parts were relatively less active, which is in agreement with their lower TPC values. Among all tested extracts, the highest ABTS (89.44 mg TE/g) and MCA (19.31 mg EDTAE/g) were shown by the water extract of aerial parts. The highest PBD value of 2.13 mmol TE/g was determined for the ethanol aerial part. In the case of root extracts, the lower antioxidant activity compared to their aerial counterparts corresponded to markedly reduced phenolic and flavonoid contents. Among the root extracts, the ethanol/water extract had the highest activity in CUPRAC (95.90 mg TE/g), ABTS (69.27 mg TE/g), FRAP (56.02 mg TE/g), and DPPH (49.84 mg TE/g), followed by the water extract in most assays. A similar pattern was reported in a previous study where aerial parts of *S. hispanica* showed stronger antioxidant activity than roots (Ak et al. 2020). The different antioxidant activities between the two organs of the studied plant may reflect differences in their phytochemical composition. For example, tricin occurs exclusively in roots, while kaempferol, quercetin, naringin, and luteolin‐*C*‐hexoside are detected in aerial parts, which could contribute to the distinct bioactivity of respective extracts (Baranowska et al. 2021).

### 3.4 Determination of Enzyme Inhibitory Properties

Table 4 shows the results of testing aerial part and root extracts of *S. parviflora* for their inhibitory activity against AChE, BChE, tyrosinase, *α*‐amylase and *α*‐glucosidase. Plants are valuable biological resources with natural anticholinesterase activity due to their rich content of phytochemicals. The results highlighted significant differences between the extracts and partial correlation with their TPC and TFC values. Among the aerial part extracts, all tested extracts except water showed almost identical inhibition of AChE and BChE at around 2 mg GALAE/g. The observed anticholinesterase activity in the extracts is most likely due to the presence of flavonoids, phenolic acids and terpenoids which are well known as AChE and BChE enzyme inhibitors (Ślusarczyk et al. 2020). This activity contributes to the preservation of acetylcholine and has therapeutic potential against neurodegenerative diseases such as Alzheimer's disease (McGleenon et al. 1999).

**TABLE 4:** Enzyme inhibitory effects of the tested extracts of *S. parviflora* .

Parts	Extracts	AChE (mg GALAE/g)	BChE (mg GALAE/g)	Tyrosinase (mg KAE/g)	Amylase (mmol ACAE/g)	Glucosidase (mmol ACAE/g)
Aerial parts	Ethyl acetate	2.15 ± 0.18^b^	2.53 ± 0.31^b^	46.53 ± 1.36^d^	0.52 ± 0.01^b^	na
	Ethanol	2.86 ± 0.08^a^	2.63 ± 0.02^b^	56.85 ± 0.56^b^	0.43 ± 0.01^c^	1.16 ± 0.01^c^
	Ethanol/Water	2.18 ± 0.08^b^	0.10 ± 0.01^d^	59.57 ± 0.27^a^	0.27 ± 0.01^e^	1.46 ± 0.01^a^
	Water	na	na	27.72 ± 0.79^e^	0.07 ± 0.01^f^	na
Roots	Ethyl acetate	1.23 ± 0.06^c^	1.20 ± 0.15^c^	51.30 ± 0.80^c^	0.57 ± 0.01^a^	na
	Ethanol	3.04 ± 0.01^a^	3.66 ± 0.35^a^	54.83 ± 0.80^b^	0.37 ± 0.01^d^	1.21 ± 0.01^b^
	Ethanol/Water	2.30 ± 0.02^b^	1.51 ± 0.27^c^	56.18 ± 0.89^b^	0.29 ± 0.01^e^	1.43 ± 0.01^a^
	Water	na	0.37 ± 0.08^d^	15.27 ± 1.38^f^	0.08 ± 0.01^f^	0.67 ± 0.03^d^

*Note:* Values are reported as mean ± SD of three parallel measurements. For each method, values followed by different letters (a–f) show significant differences at *p <* 0.05 (Tukey's test).

Abbreviations: ACAE, Acarbose equivalent; GALAE, Galantamine equivalent; KAE, Kojic acid equivalent; na, not active.

Another studied enzyme, tyrosinase, is a rate‐limiting enzyme in melanin biosynthesis that catalyzes pigmentation of skin and hair. Overexpression of this enzyme results in hyperpigmentation disorders such as melasma, age spots, and actinic damage (Zolghadri et al. 2019). Among the extracts from the aerial part, ethanol/water extract showed the highest inhibition against tyrosinase with 59.57 mg KAE/g followed by ethanol extract with 56.85 mg KAE/g, indicating dermoprotective effect of *S. parviflora* . The activity against tyrosinase may be attributed to the presence of flavonoids, Quercetin (Fan et al. 2017) and kaempferol‐*O*‐galloylrhamnoside (Rho et al. 2011). However, water extract was the least active in all assays. Among the root's extracts, ethanol extract exhibited the most potent dual inhibitory activity against AChE with 3.04 mg GALAE/g and BChE with 3.66 mg GALAE/g, along with moderate inhibition against tyrosinase with 54.83 mg KAE/g and *α*‐glucosidase with 1.21 mmol ACAE/g. Ethanol/water extract also had relatively high antityrosinase activity with 56.18 mg KAE/g. Previous studies confirmed the enzyme inhibitory activities of *Scorzonera* genus, supporting its potential as a source of bioactive compounds (Ak et al. 2020; Dall'Acqua, Ak, Sut, Ferrarese, et al. 2020; Deveci 2022). All extracts were less or not active against carbohydrate digestive enzymes (*α*‐amylase and *α*‐glucosidase).

These findings are in agreement with our previous work showing that phenolic‐rich natural extracts often exhibit potent and relevant bioactivities (El‐Nashar et al. 2021). Overall, the *S. parviflora* extracts rich in phenolic and flavonoid compounds, particularly ethanol and ethanol/water extracts, exhibited higher enzyme inhibitory activities, indicating a positive correlation between them. The results suggest that phenolic constituents may therefore contribute significantly to the observed enzyme inhibitory potential of *S. parviflora* .

### 3.5 Cytotoxic Potential

Hepatocellular carcinoma (HCC) and neuroblastoma represent two highly malignant tumors (Chen et al. 2025). As the most prevalent primary liver cancer, HCC exhibits rapid disease progression and high metastatic/recurrent potential, thereby establishing it as a leading cause of cancer‐related mortality globally (Hou et al. 2025). Neuroblastoma, originating from the sympathetic nervous system, constitutes the most common extracranial solid malignancy in pediatric populations; its pronounced biological heterogeneity and aggressive clinical behavior present significant therapeutic challenges (Li, Gu, Tang, et al. 2025). Although conventional treatment modalities, including cytotoxic chemotherapy, molecular targeted therapies, and radiotherapeutic interventions, demonstrate limited efficacy against these malignancies, they are often accompanied by severe systemic adverse effects, including bone marrow suppression, hepatorenal functional impairment, and neurological toxicity, which substantially diminish both patients' quality of life and long‐term survival outcomes (Li, Gu, Li, et al. 2025). Consequently, the systematic identification and preclinical development of naturally derived phytochemicals with favorable safety profiles that demonstrate robust antitumor activity, coupled with mechanistic elucidation of their molecular modes of action, possess substantial translational significance for optimizing adjuvant therapeutic regimens or establishing alternative treatment paradigms to enhance clinical management of these malignancies (Li, Zhang, et al. 2025).

The cytotoxic potential of *S. parviflora* extracts from aerial parts and roots was evaluated in three human cell lines: HEK 293 (embryonic kidney), HepG2 (hepatocellular carcinoma), and SH‐SY5Y (neuroblastoma; Table 5). In general, polar extracts (EtOH/Water and Water) showed high cell viability, with the hydroalcoholic extract from aerial parts being the least cytotoxic for all cell lines (70.1%–105.8%) followed by the aqueous extract (80.5%–83.4%). A similar result was observed for root extracts, where both hydroalcoholic and aqueous fractions maintained cell viability above 73%. On the contrary, ethyl acetate extracts were the most cytotoxic ones, especially those obtained from roots, which reduced viability to values as low as 5.7%–10.3%. The ethanolic extracts presented a variable response depending on the plant organ and cell line: while the aerial part ethanol extract moderately decreased the viability of HepG2 and SH‐SY5Y cells (50%–70%), the root ethanol extract caused a more pronounced effect, with viability values below 40% in most cases.

**TABLE 5:** Cytotoxicity of the extracts from *S. parviflora* on HEK 293, HepG2, and SHSY5Y cell lines (at least three experiments performed in triplicate; *n* = 9).

Parts	Extracts	HEK 293	HepG2	SHSY5Y
Aerial parts	Ethyl acetate	12.77 ± 4.33	27.27 ± 5.7	46.49 ± 6.47
	EtOH	17.08 ± 4.8	52.7 ± 5.57	67.6 ± 5.87
	EtOH/Water	105.78 ± 1.97	70.12 ± 6.21	87.56 ± 6.76
	Water	80.46 ± 7.39	63.92 ± 6.68	83.35 ± 3.86
Roots	Ethyl acetate	10.26 ± 3.98	8.3 ± 0.73	5.69 ± 0.56
	EtOH	10.4 ± 3.24	37.83 ± 3.8	61.29 ± 4.91
	EtOH/Water	77.13 ± 6.61	92.76 ± 2.04	91.56 ± 6.27
	Water	86.97 ± 4.39	73.29 ± 5.1	91.33 ± 4.49

*Note:* Extracts were tested at 100 μg/mL, and results are expressed as a percentage of cellular viability (%) relative to the control containing 0.5% DMSO. Values represent the mean ± standard error of the mean (SEM).

According to the ISO 10993‐5 classification (ISO 10993‐5: 2009 2009), cell viability above 80% indicates non‐cytotoxicity, 60%–80% weak, 40%–60% moderate, and below 40% strong cytotoxicity. In this study, hydroethanolic and aqueous extracts of *S. parviflora* maintained cell viability mostly above 70%–80%, suggesting low or no cytotoxic effects at tested concentrations.

Similar patterns have been described for other *Scorzonera* species (Lendzion et al. 2021). Assays on *S. hispanica* , *S. divaricata* , *S. austriaca*, and *S. mollis subsp. szowitzii* showed that non‐polar extracts (hexane, chloroform, or ethyl acetate) exerted moderate to strong cytotoxicity (IC_50_ = 5–95 μg/mL) against human cancer cell lines such as HeLa, MCF‐7, A549, PC‐3, HepG2, HT‐29, and U87, whereas polar fractions (methanol, hydroalcoholic, or aqueous extracts) were generally weakly or not cytotoxic, maintaining viability above 70%–80% in HepG2, C6, and HT‐29 cells, and were also biocompatible with non‐tumoral Vero and NIH/3 T3 cells. Similarly low toxicity was observed by Deveci et al. for *S. mollis subsp. szowitzii*, where the hydroalcoholic extract had negligible effects on cell viability (Deveci 2022; Wu et al. 2026).

The LC–MS analysis of *S. parviflora* extracts supports the observed biological trends. The hydro‐alcoholic and aqueous extracts, especially those from the aerial parts, were rich in hydrophilic phenolic acids (quinic, chlorogenic, caffeoylquinic, and dicaffeoylquinic acids), coumarins (umbelliferone, scopoletin), and flavonoids or their glycosides (naringenin derivatives, luteolin‐*C*‐hexoside, kaempferol, tricin, and isorhamnetin glycoside). These classes of compounds have been mainly associated with antioxidant and anti‐inflammatory rather than cytotoxic effects in *Scorzonera* spp. (Lendzion et al. 2021; Deveci 2022). On the other hand, the ethyl acetate and ethanol fractions contained less polar constituents such as sesquiterpenes, triterpenoids, and lipid derivatives, which have been linked to higher levels of cytotoxicity in other *Scorzonera* spp. (Lendzion et al. 2021).

Overall, the results show that solvent polarity affects both chemical composition and cytotoxic behavior of *S. parviflora* extracts, with the highest biocompatibility under the tested conditions being shown by the polar fractions rich in phenolic acids, flavonoids, and coumarins.

### 3.6 Molecular Docking

The selection of the 11 target compounds for subsequent molecular docking and network pharmacology analyses was strategically guided by three complementary lines of evidence from our experimental data. First, we prioritized compounds with the highest quantitative abundance across the various solvent extracts, as these represent the dominant phytochemical constituents most likely responsible for the observed bioactivities. Second, we focused on compounds exhibiting significant tissue‐specific distribution differences between the aerial parts and roots, or between polar and non‐polar fractions, to capture the structural diversity underlying the spatial variation in efficacy. Third, the selection was informed by the solvent‐dependent extraction efficiency of these compounds, aligning with the corresponding trends in in vitro antioxidant and enzyme inhibitory activities, thereby enabling a direct linkage between chemical profiles and functional properties.

**FIGURE 1:**
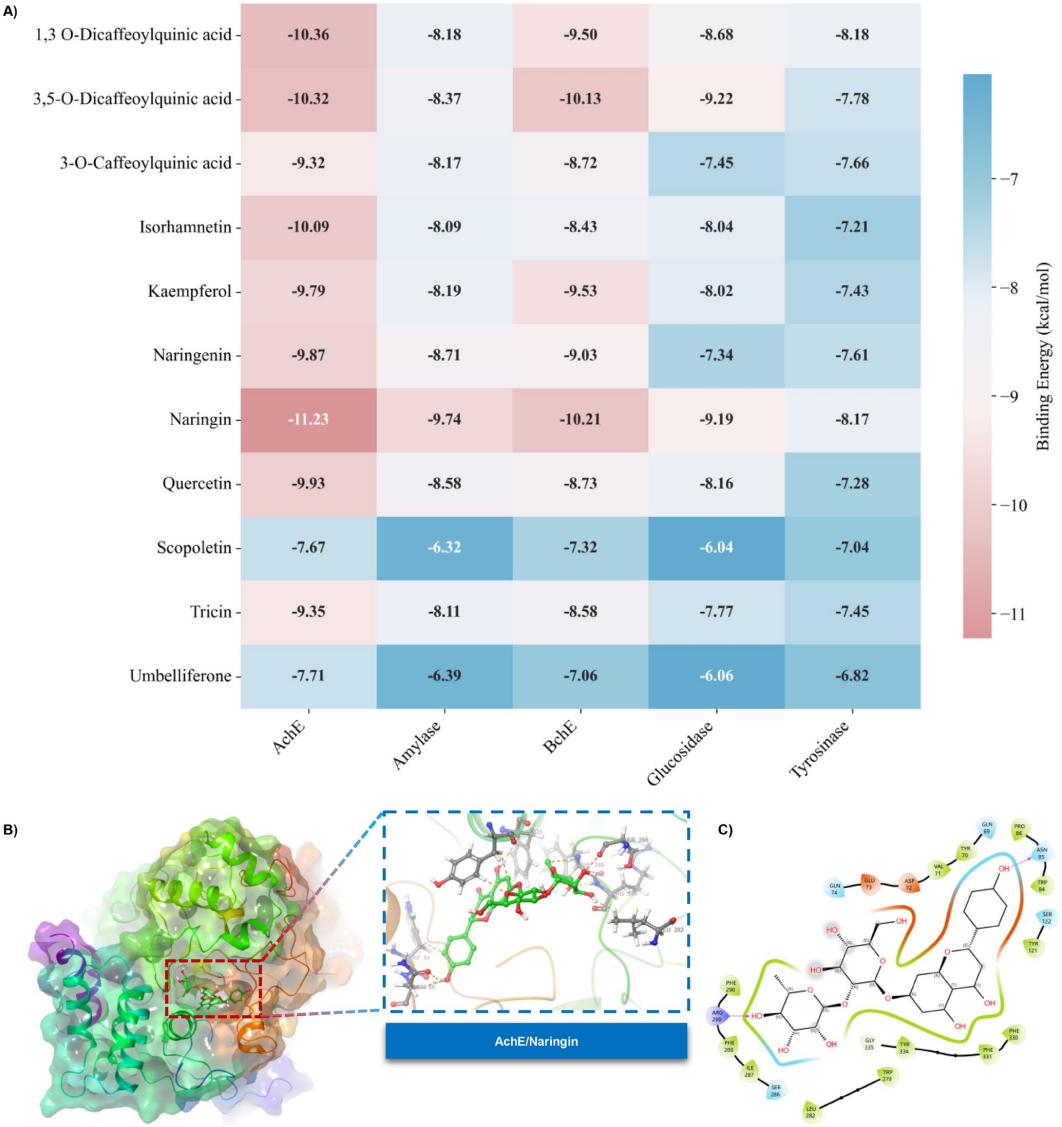
The docking results and correlation analysis of 11 compounds from *S. parviflora* against 5 metabolic enzymes. (A) Docking Δ*G* heat‐map (kcal/mol). (B) Binding mode of naringin in the AChE active site. (C) The 2D representation of the binding pockets of AChE with naringin.

Molecular docking analysis of 11 compounds from *S. parviflora* extracts against five enzyme targets demonstrated significant binding affinities for all tested compounds, with binding energies below −6.0 kcal/mol suggesting potential inhibitory activities. Among these compounds, naringin exhibited the most potent binding interactions across all target enzymes (AChE: −11.23; BChE: −10.21; amylase: −9.74; glucosidase: −9.19; tyrosinase: −8.17). Caffeoylquinic acid derivatives including 1,3‐*O*‐dicaffeoylquinic acid and 3,5‐*O*‐dicaffeoylquinic acid, that were previously identified in *Scorzonera*, along with flavonoids such as quercetin and kaempferol, also displayed strong multi‐target binding capacities. In contrast, coumarin derivatives including scopoletin and umbelliferone exhibited comparatively weaker binding affinities (Figure 1).

This study demonstrates through molecular docking that several *S. parviflora* constituents exhibit multi‐target enzyme inhibitory potential, with flavonoid glycoside naringin being particularly prominent. AChE and BChE represent key therapeutic targets for neurodegenerative disorders like Alzheimer's disease, while amylase and glucosidase participate in blood glucose homeostasis regulation in type 2 diabetes mellitus. Tyrosinase serves as a critical mediator of melanogenesis and related dermatological pathologies. The observed high binding affinities suggest potential applications of this plant in neuroprotection, hyperglycemia management, and hyperpigmentation disorders. Notably, multiple compounds displayed strong binding interactions with both AChE and BChE, with naringin exhibiting binding energies below −10.0 kcal/mol for both cholinesterases. This dual cholinesterase inhibition profile highlights its potential as a promising candidate for developing multi‐target directed ligands in neuroprotective therapy. The significant inhibitory activities observed in caffeoylquinic acids and flavonoids may originate from structural features including phenolic hydroxyl groups, conjugated systems, and glycosylation patterns that facilitate stable enzyme active site interactions.

### 3.7 Network Pharmacology

**FIGURE 2:**
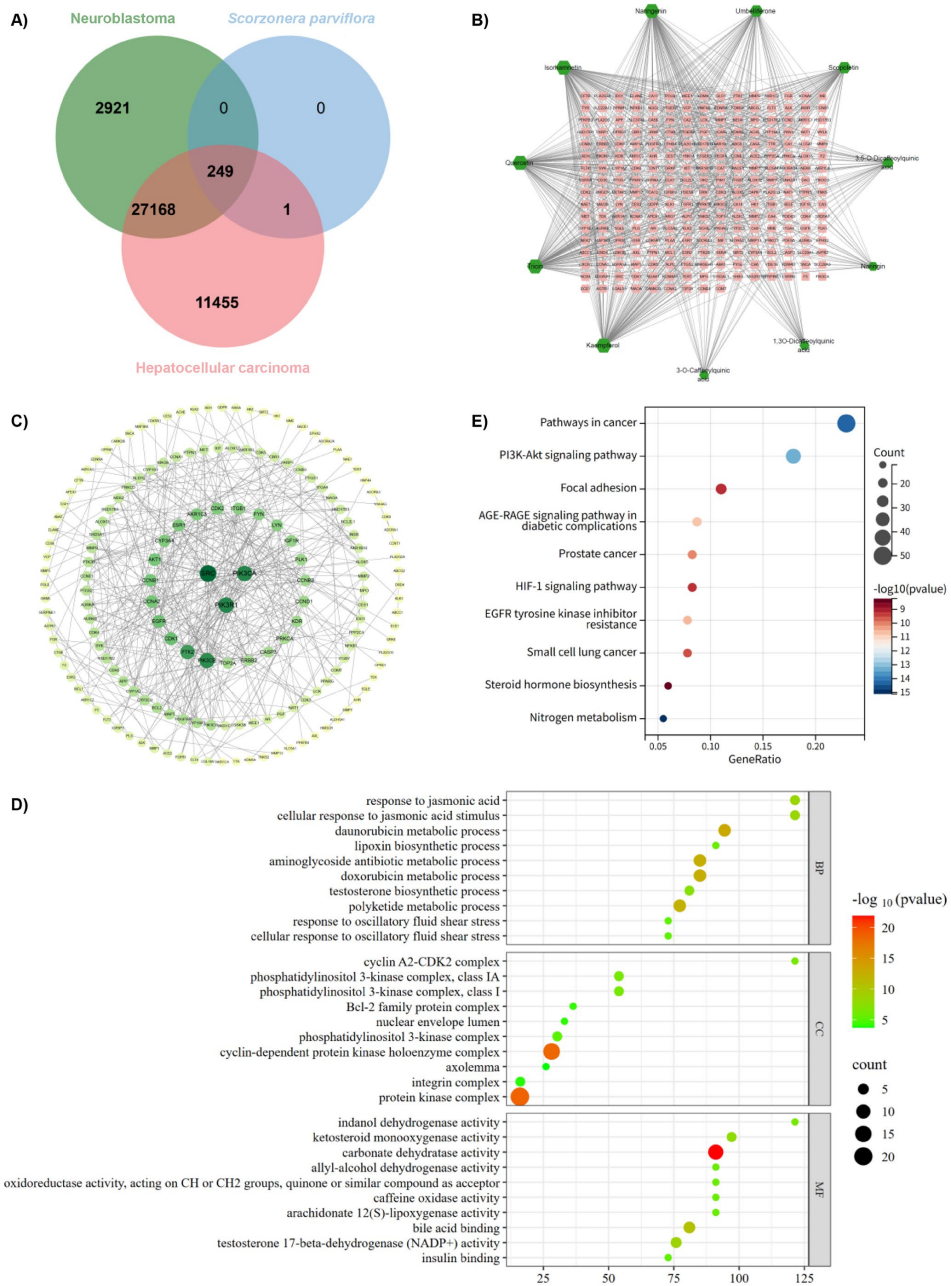
Network pharmacology and functional enrichment analysis of the *S. parviflora* extracts in neuroblastoma and hepatocellular carcinoma. (A) The Venn diagram shows 249 common targets of compounds from the *S. parviflora* extracts in neuroblastoma and hepatocellular carcinoma. (B) The active compounds‐targets network in neuroblastoma and hepatocellular carcinoma. (C) The PPI network of 249 co‐targeted genes/proteins. (D) The GO enrichment analysis results for the anti‐cancer targets. (E) The top 10 KEGG pathways enriched.

In order to explore the potential therapeutic mechanisms of *S. parviflora* in combating neuroblastoma and hepatocellular carcinoma, we utilized a network pharmacology approach. The potential targets of 11 active compounds derived from *S. parviflora* were identified using the SwissTargetPrediction database. Concurrently, disease‐related targets for neuroblastoma and hepatocellular carcinoma were sourced from reputable databases such as GeneCards, DrugBank, OMIM, TTD, and CTD. An intersection analysis of these target groups yielded 249 targets that were common to the compound targets and both disease targets (Figure 2A). One unique target was shared between the compound targets and hepatocellular carcinoma targets. These overlapping targets are considered key for the synergistic anti‐tumor effects of *S. parviflora* . Further analysis identified 11 core active compounds associated with the 249 common targets: naringenin, umbelliferone, scopoletin, 3,5‐*O*‐dicaffeoylquinic acid, naringin, 1,3‐*O*‐dicaffeoylquinic acid, 3‐*O*‐caffeoylquinic acid, kaempferol, tricin, quercetin, and isorhamnetin. The interaction network relationship between these compounds and their corresponding targets is summarized in Figure 2B, which reveals a complex mechanism by which *S. parviflora* exerts its potential therapeutic effects on both cancers through a “multi‐component, multi‐target” model.

To further explore the interaction among these 249 common targets, a protein–protein interaction (PPI) network with 167 nodes and 424 edges was constructed. In this network (Figure 2C), the color and size of each node were determined by its degree centrality value, which ranged from green to yellow gradient and large to small gradient, respectively. The nodes with larger size and green color represented higher values of degree centrality. This visual method intuitively showed the topological importance of each protein in the network. Further topological analysis identified several core hub genes, including SRC, PIK3CA, PIK3R1, PIK3CB, PTK2, CDK1, and EGFR. These genes act as key nodes in the network, indicating that they may be important regulatory factors in the pathways through which *S. parviflora* exerts therapeutic effects against neuroblastoma and hepatocellular carcinoma, thereby reflecting their biological significance.

GO functional enrichment analysis systematically demonstrated that *S. parviflora* modulates core cancer pathways (Figure 2D). The findings revealed significant enrichment of its core targets in Biological Processes (BP) such as the “daunorubicin/doxorubicin metabolic process,” “lipoxin biosynthetic process,” and “response to jasmonic acid.” This suggests that the active compounds might influence chemotherapeutic drug metabolism, regulate inflammation within the tumor microenvironment, and directly induce cellular stress responses. Cellular Component (CC) analysis localized these functions to pivotal cancer signaling hubs, including the “phosphatidylinositol 3‐kinase (PI3K) complex,” “cyclin A2‐CDK2 complex,” and “Bcl‐2 family protein complex.” Molecular Function (MF) analysis further elucidated these processes with terms like “arachidonate 12(S)‐lipoxygenase activity” and “insulin binding,” both integral to signaling pathways like PI3K. In essence, the active compounds target key signaling complexes, notably PI3K and CDK, disrupting their enzymatic activities and binding capacities, leading to a synergistic inhibition of vital macroscopic processes such as tumor cell metabolism, proliferation, and apoptosis.

To elucidate the mechanism of action at a systematic level, KEGG pathway enrichment analysis was performed on core targets (Figure 2E). The results showed that the targets were highly enriched in core pathways regulating cell proliferation and survival, with the PI3K‐Akt signaling pathway, Ras signaling pathway, and MAPK signaling pathway being particularly significant, all of which are key components of the overarching “Pathways in cancer”. Meanwhile, the enrichment of the “Focal adhesion” pathway confirmed its potential to inhibit tumor invasion and metastasis. Importantly, the significantly enriched “Hepatitis B” pathway provided a strong etiological link for the therapeutic mechanism of drugs against hepatocellular carcinoma. In conclusion, the KEGG analysis demonstrated that *S. parviflora* exerted potent synergistic antitumor effects by simultaneously blocking core growth signals, interfering with cellular adhesion processes, and targeting disease‐specific pathways.

## 4 Conclusion

Interest in plant bioactives has increased due to growing concerns about the side effects of synthetic drugs. This study investigated the phytochemical composition and bioactive potential of *S. parviflora* , highlighting its significance as a natural food additive and medicinal resource. Among the tested extracts, the water and ethanol/water extracts from the aerial parts exhibited the highest TPC values, whereas the EA extract contained the highest flavonoid concentration. antioxidant activity varied significantly, generally correlating with the observed levels of TPC and TFC. Among the aerial part extracts, the ethanol/water and water extracts demonstrated the highest antioxidant capacity across most assays. All aerial extracts except the water extract displayed comparable AChE and BChE inhibitory activities, while the ethanol/water extract showed the strongest tyrosinase inhibition, likely attributable to flavonoids and phenolic compounds such as quercetin and kaempferol‐*O*‐galloylrhamnoside. The broad chemical profile combined with antioxidant and enzyme inhibitory activities suggests promising therapeutic and dermoprotective potential for these extracts, indicating their value in developing medicinal formulations and natural plant‐based cosmetic or pharmaceutical products. Further studies are required to identify the compounds responsible for the observed biological activities and to determine their pharmacokinetic and toxic properties in vivo.

## Author Contributions

**Maria João Rodrigues:** conceptualization, methodology, writing – review and editing, writing – original draft, data curation. **Milena Terzic:** conceptualization, methodology, writing – original draft, writing – review and editing, investigation. **Gokhan Zengin:** conceptualization, investigation, methodology, validation, writing – review and editing, writing – original draft, supervision. **Nilofar Nilofar:** conceptualization, methodology, investigation, writing – review and editing. **Meng‐Yao Li:** conceptualization, investigation, writing – original draft, writing – review and editing, supervision. **Evren Yildiztugay:** conceptualization, resources, writing – review and editing, writing – original draft, validation. **Gunes Ak:** conceptualization, data curation, investigation, writing – original draft, writing – review and editing. **Omayma A. Eldahshan:** conceptualization, investigation, writing – original draft, methodology, software. **Abdel Nasser Singab:** supervision, conceptualization, writing – review and editing, investigation. **Shaza Aly:** conceptualization, investigation, methodology, validation, writing – review and editing, writing – original draft. **Eliana Fernandes:** conceptualization, methodology, writing – original draft, writing – review and editing, investigation. **Luísa Custódio:** conceptualization, supervision, methodology, writing – original draft, writing – review and editing, investigation.

## Funding

The authors have nothing to report.

## Ethics Statement

The authors have nothing to report.

## Consent

The authors have nothing to report.

## Conflicts of Interest

The authors declare no conflicts of interest.

## Supporting information


**Figure S1:** Total ion chromatogram (TIC) of the aerial parts ethyl acetate extract of *parviflora* in negative ion mode (A) and positive ion mode (B).
**Figure S2:** Total ion chromatogram (TIC) of the aerial parts ethanol extract of S. parviflora in negative ion mode (A) and positive ion mode (B).
**Figure S3:** Total ion chromatogram (TIC) of the aerial parts ethanol/water extract of S. parviflora in negative ion mode (A) and positive ion mode (B).
**Figure S4:** Total ion chromatogram (TIC) of the aerial parts water extract of S. parviflora in negative ion mode (A) and positive ion mode (B).
**Figure S5:** Total ion chromatogram (TIC) of the roots ethyl acetate extract of S. parviflora in negative ion mode (A) and positive ion mode (B).
**Figure S6:** Total ion chromatogram (TIC) of the roots ethanol extract of S. parviflora in negative ion mode.
**Figure S7:** Total ion chromatogram (TIC) of the roots ethanol/water extract of S. parviflora in positive ion mode.
**Figure S8:** Total ion chromatogram (TIC) of the roots water extract of S. parviflora in negative ion mode (A) and positive ion mode (B).

## Data Availability

The data that support the findings of this study are available on request from the corresponding author.
