# Supplementary material for: From Metabolites to Mechanisms: Scorzonera parviflora Aerial Parts and Roots Extracts Profiled by UPLC‐ESI‐MS/MS, In Vitro/In Silico Tests, and Network Analysis
Source: Food Sci Nutr. 2026 Jul 9;14(7):e72092. doi: 10.1002/fsn3.72092 (PMC13347232; doi:10.1002/fsn3.72092)
Supplement: Supplementary file 1 — Figure S1: Total ion chromatogram (TIC) of the aerial parts ethyl acetate extract of parviflora in negative ion mode (A) and positive ion mode (B). Figure S2: Total ion chromatogram (TIC) of the aerial parts ethanol extract of S. parviflora in negative ion mode (A) and positive ion mode (B). Figure S3: Total ion chromatogram (TIC) of the aerial parts ethanol/water extract of S. parviflora in negative ion mode (A) and positive ion mode (B). Figure S4: Total ion chromatogram (TIC) of the aerial parts water extract of S. parviflora in negative ion mode (A) and positive ion mode (B). Figure S5: Total ion chromatogram (TIC) of the roots ethyl acetate extract of S. parviflora in negative ion mode (A) and positive ion mode (B). Figure S6: Total ion chromatogram (TIC) of the roots ethanol extract of S. parviflora in negative ion mode. Figure S7: Total ion chromatogram (TIC) of the roots ethanol/water extract of S. parviflora in positive ion mode. Figure S8: Total ion chromatogram (TIC) of the roots water extract of S. parviflora in negative ion mode (A) and positive ion mode (B). [file FSN3-14-e72092-s001.docx]

**From metabolites to mechanisms: *Scorzonera parviflora* aerial parts and roots extracts profiled by UPLC-ESI-MS/MS, *in vitro*/*in silico* tests, and network analysis**

| **A**  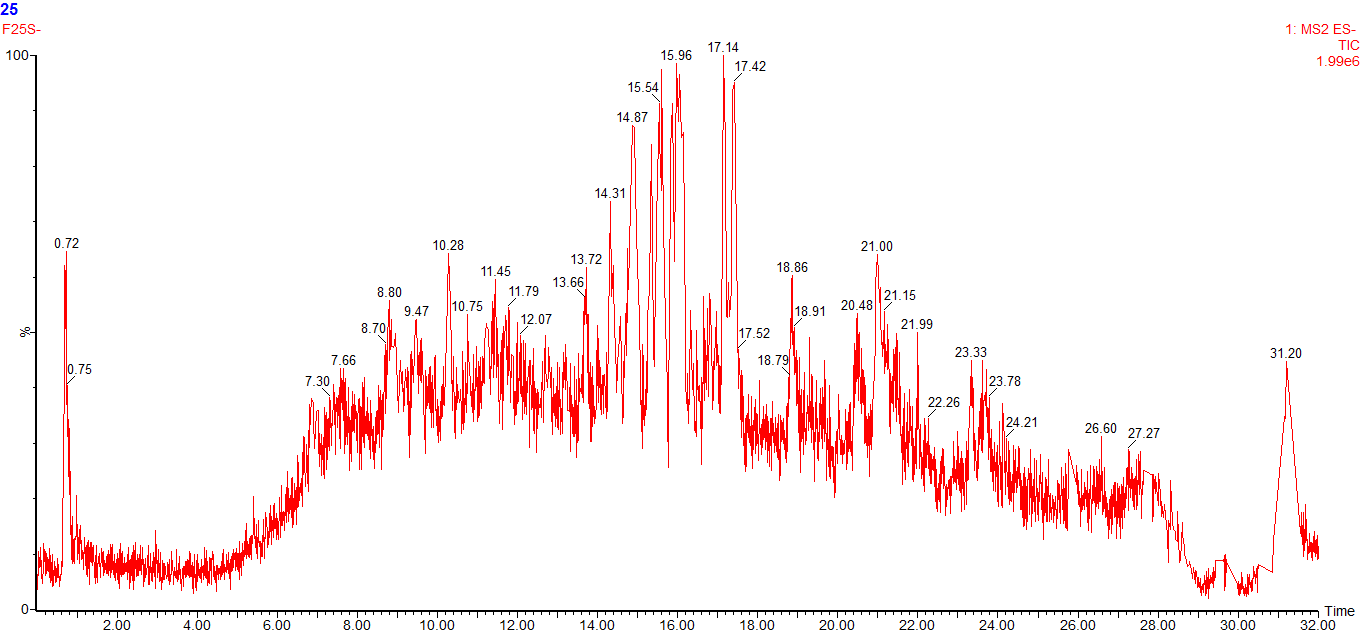 |
| --- |
| **B**  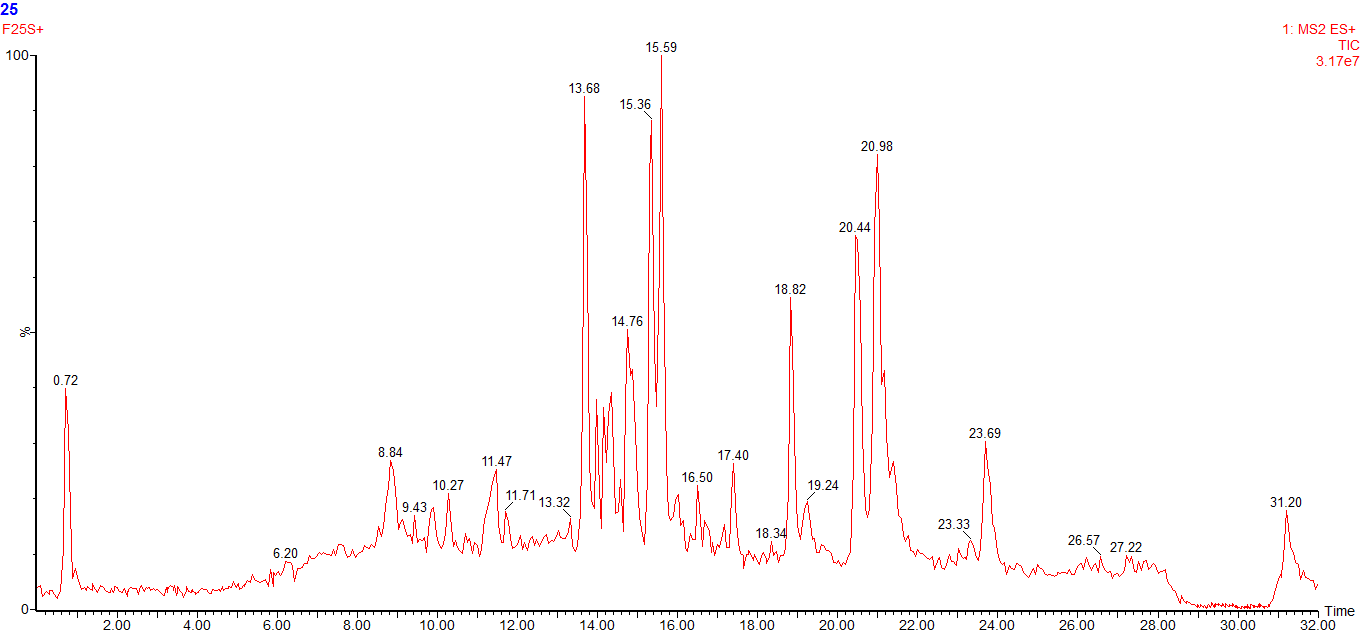 |

**Figure 1. Total ion chromatogram (TIC) of the aerial parts ethyl acetate extract of *parviflora* in negative ion mode (A) and positive ion mode (B)**

| **A**  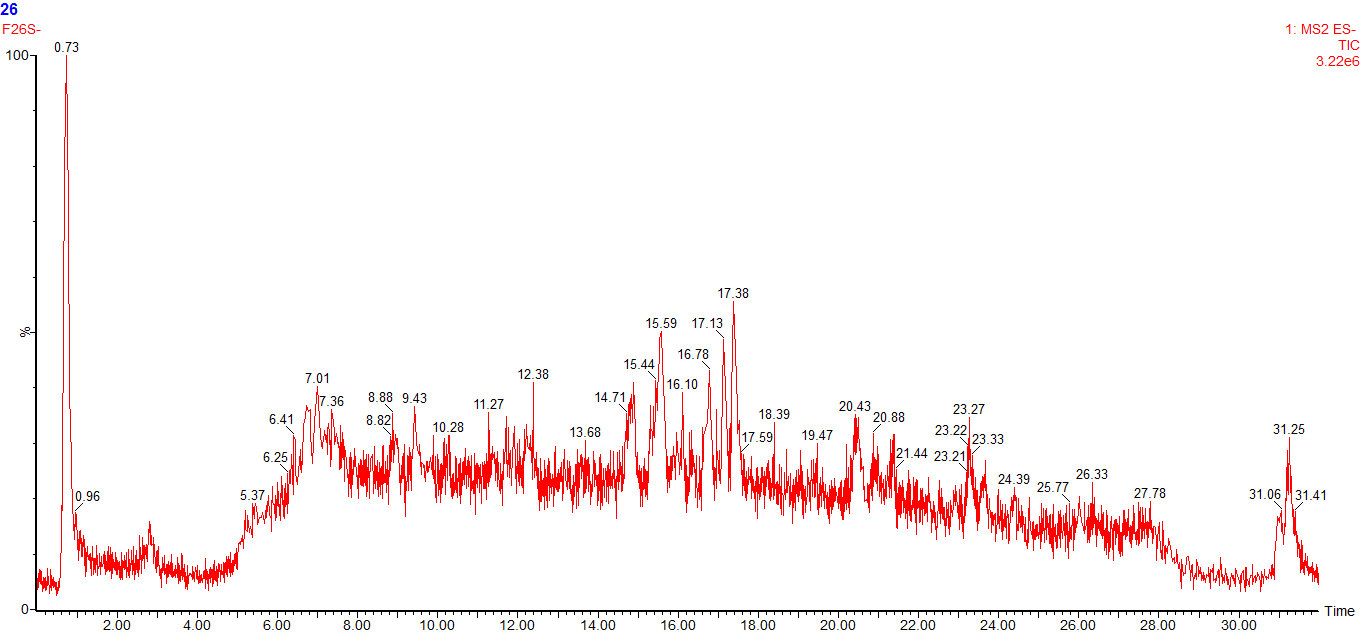 |
| --- |
| **B**  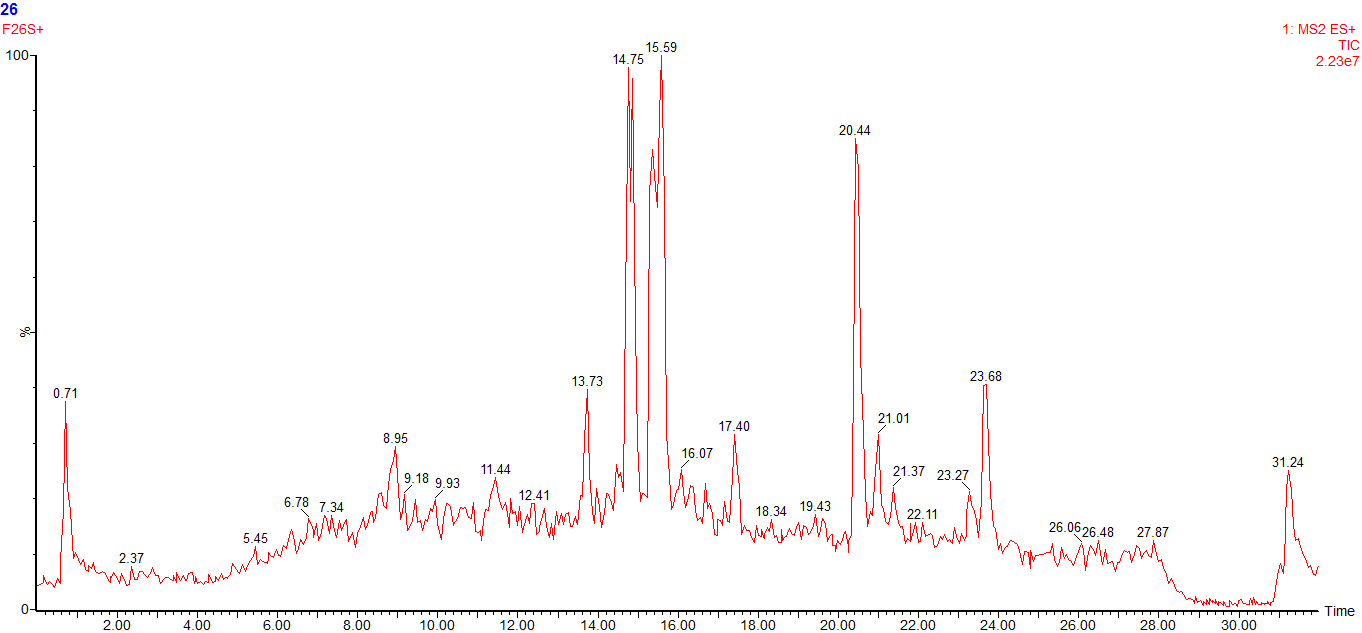 |

**Figure 2. Total ion chromatogram (TIC) of the aerial parts ethanol extract of *S. parviflora* in negative ion mode (A) and positive ion mode (B)**

| **A**  **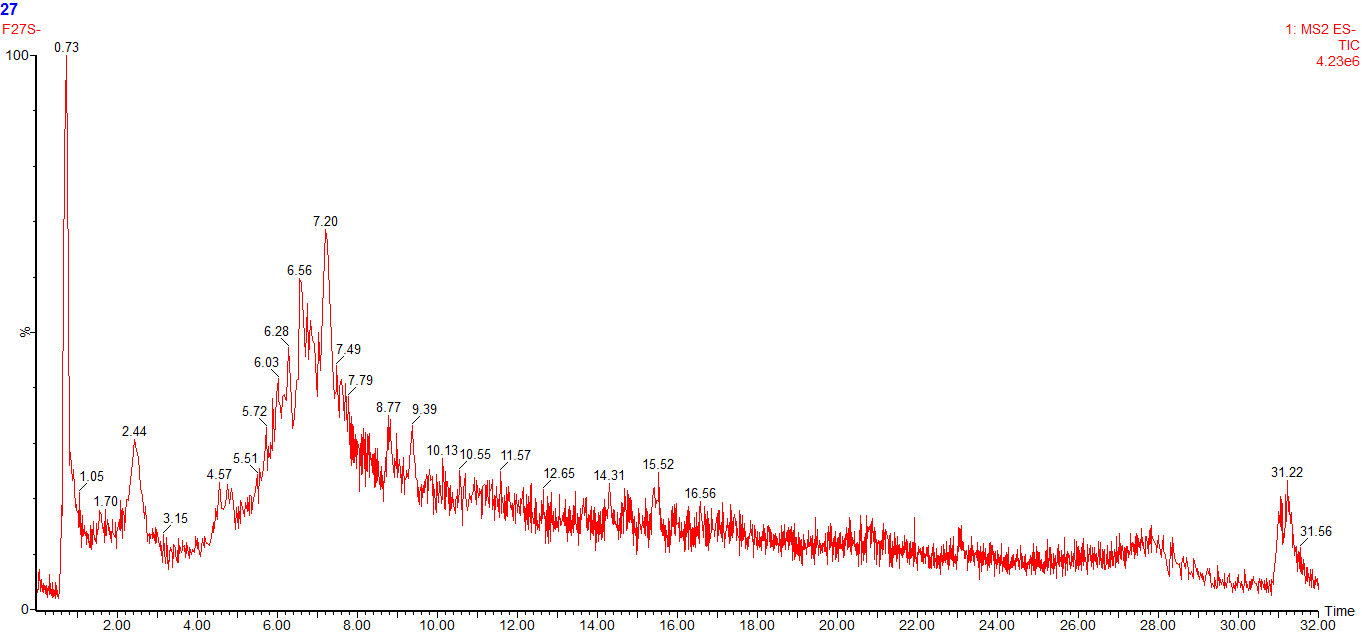** |
| --- |
| **B**  **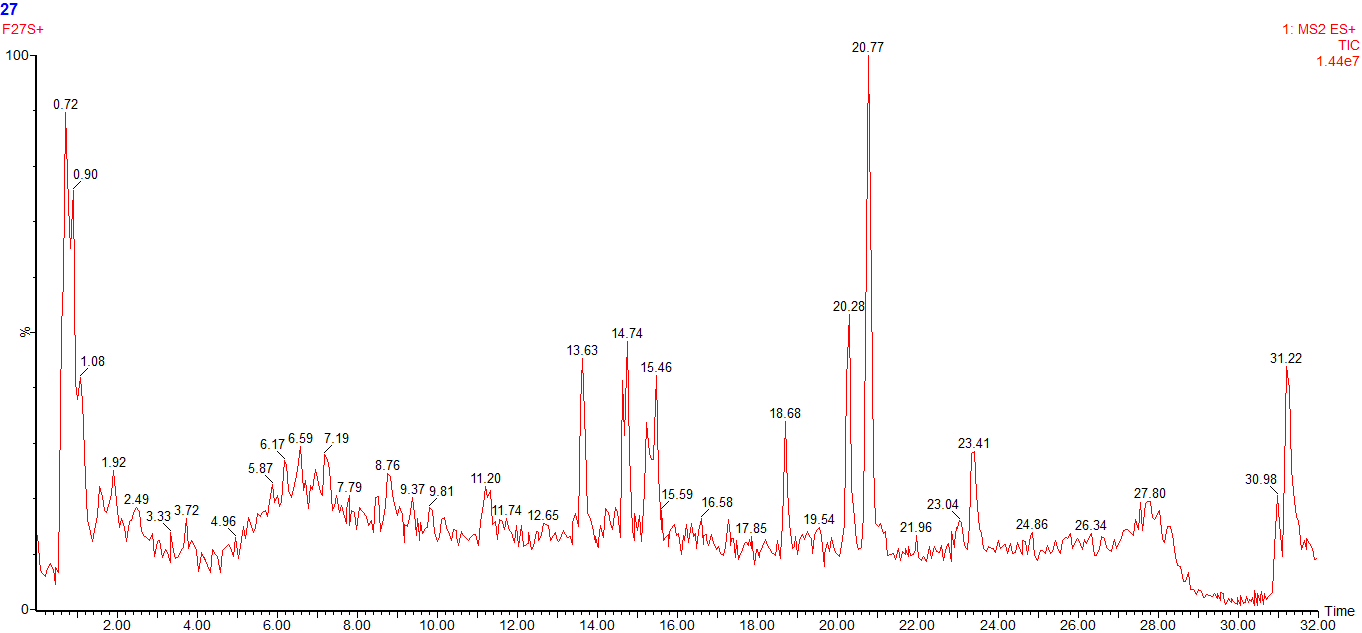** |

**Figure S3. Total ion chromatogram (TIC) of the aerial parts ethanol/water extract of *S. parviflora* in negative ion mode (A) and positive ion mode (B)**

| **A**  **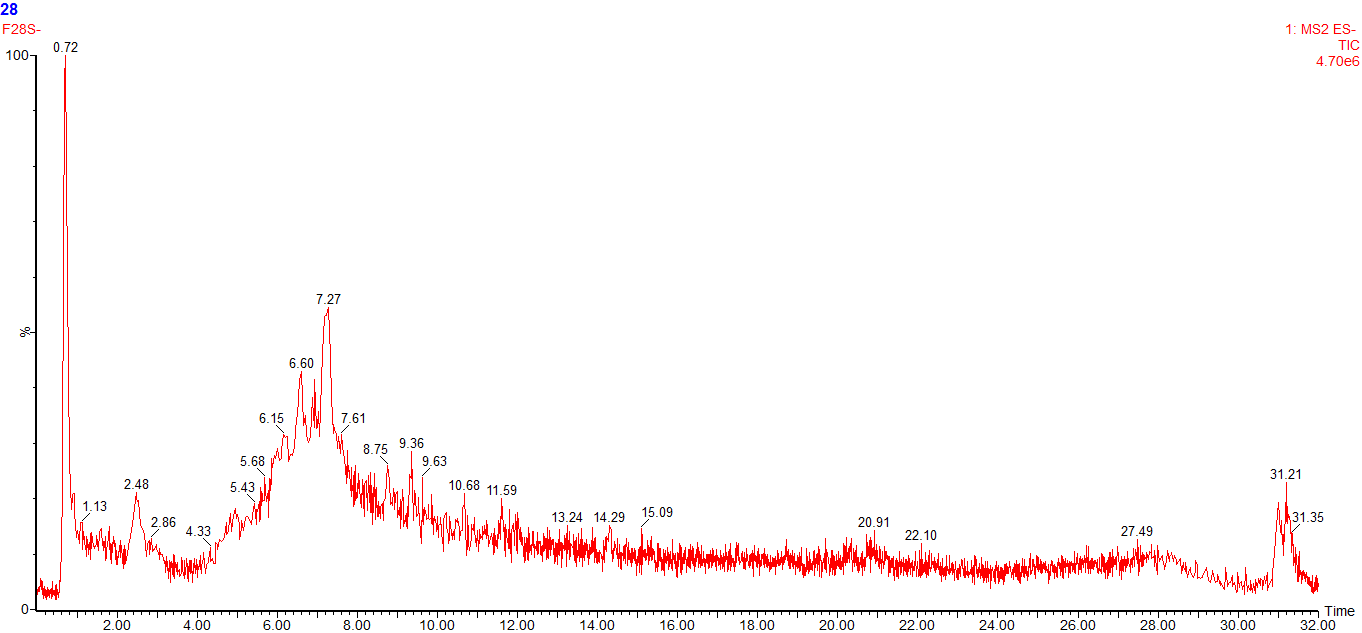** |
| --- |
| **B**  **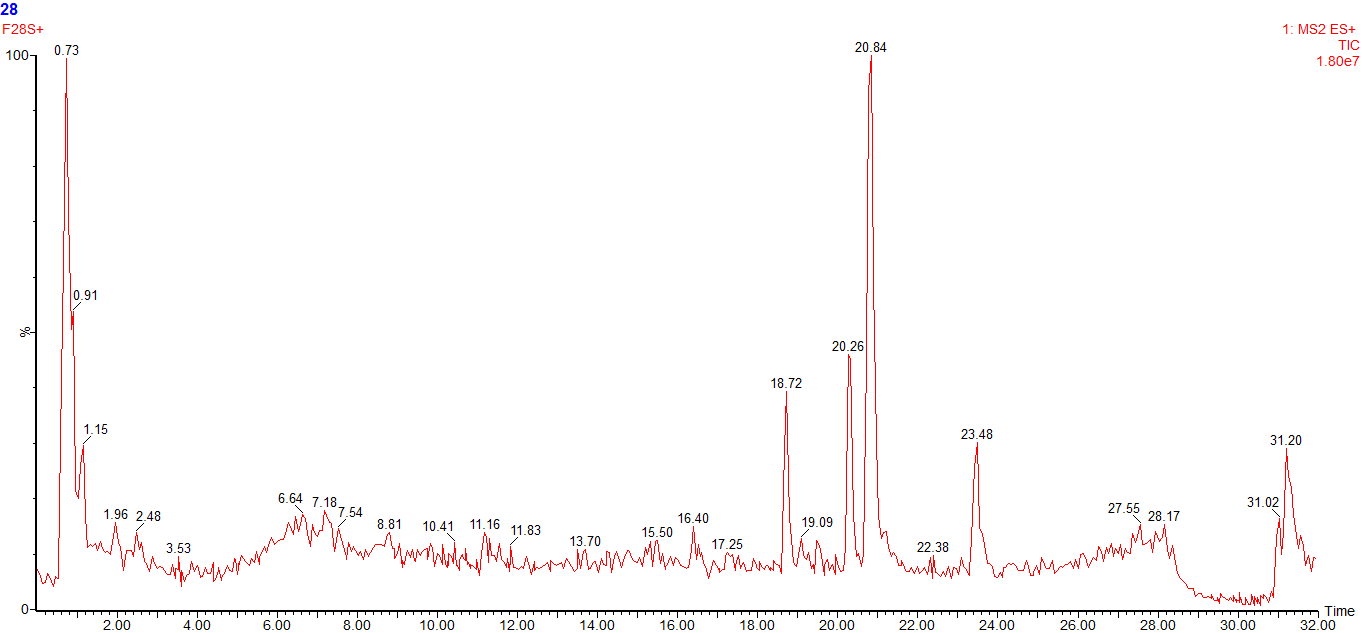** |

**Figure S4. Total ion chromatogram (TIC) of the aerial parts water extract of *S. parviflora* in negative ion mode (A) and positive ion mode (B)**

| **A**  **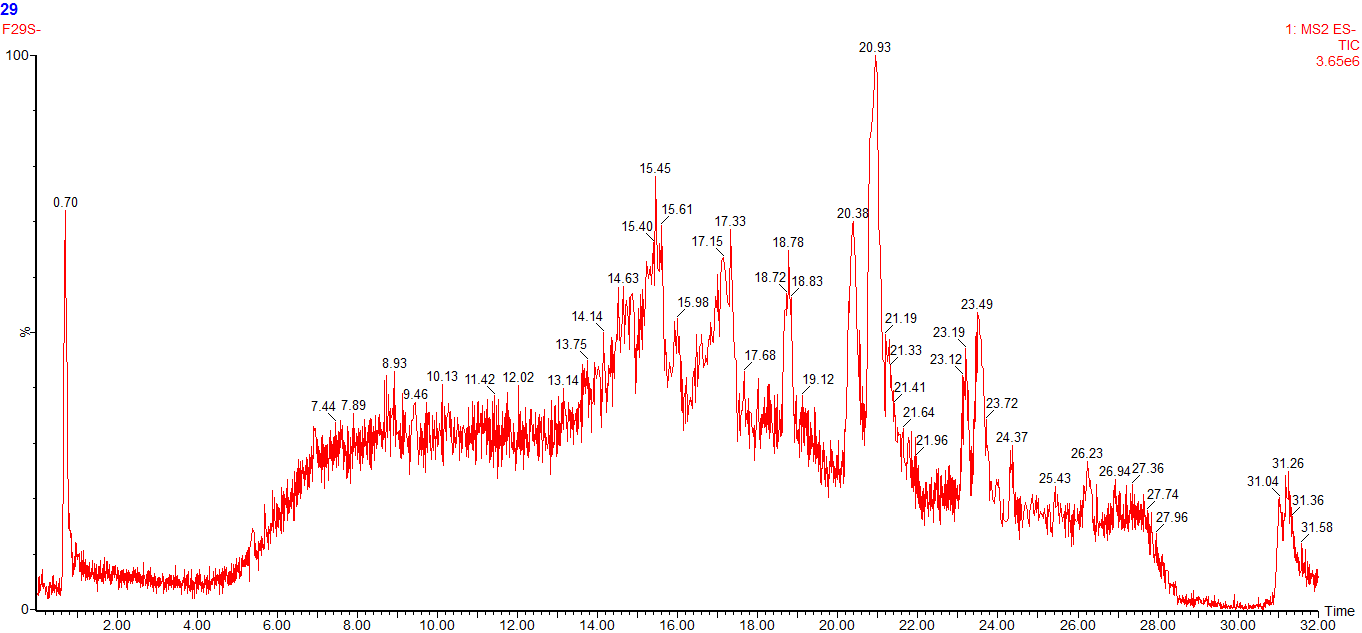** |
| --- |
| **B**  **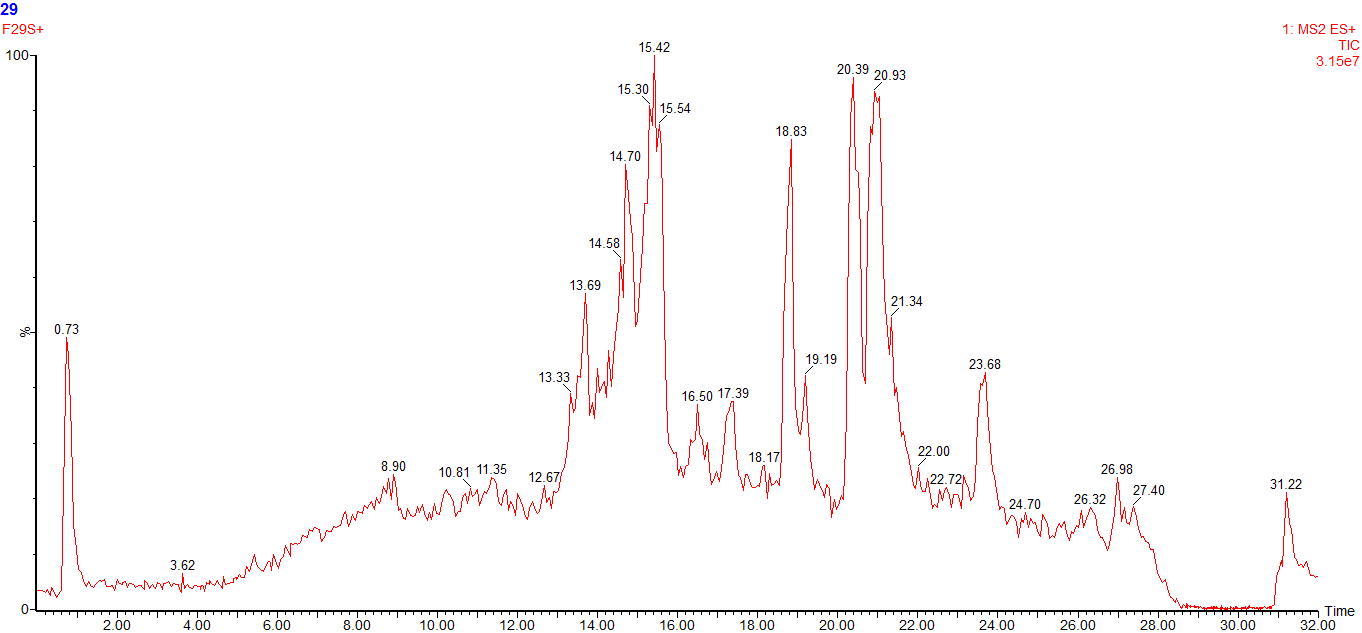** |

**Figure S5. Total ion chromatogram (TIC) of the roots ethyl acetate extract of *S. parviflora* in negative ion mode (A) and positive ion mode (B)**

| **A**  **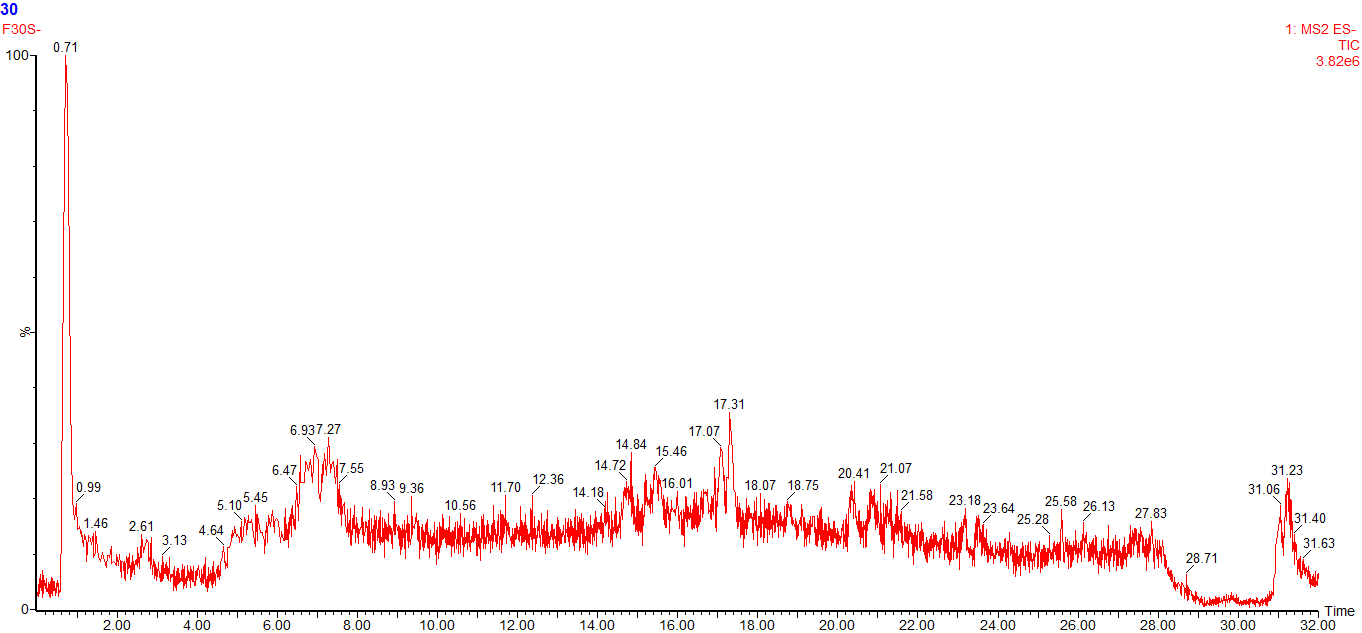** |
| --- |

**Figure S6. Total ion chromatogram (TIC) of the roots ethanol extract of *S. parviflora* in negative ion mode**

| **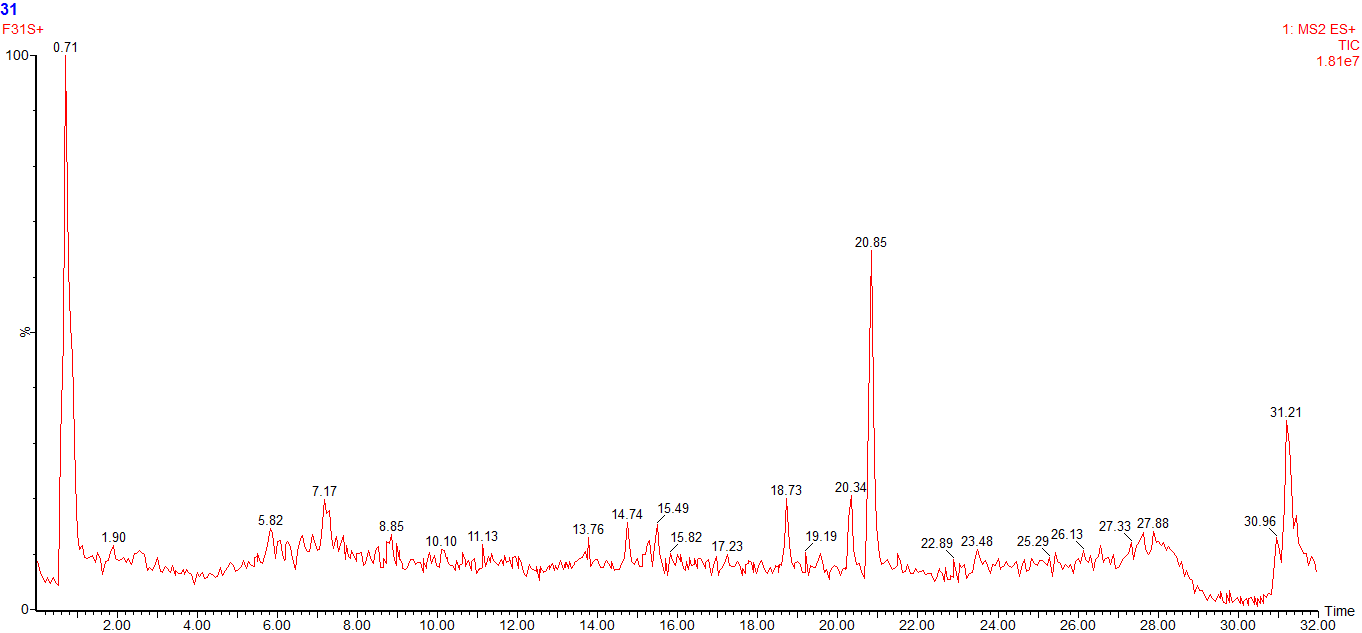** |
| --- |

**Figure S7. Total ion chromatogram (TIC) of the roots ethanol/water extract of *S. parviflora* in positive ion mode**

| **A**  **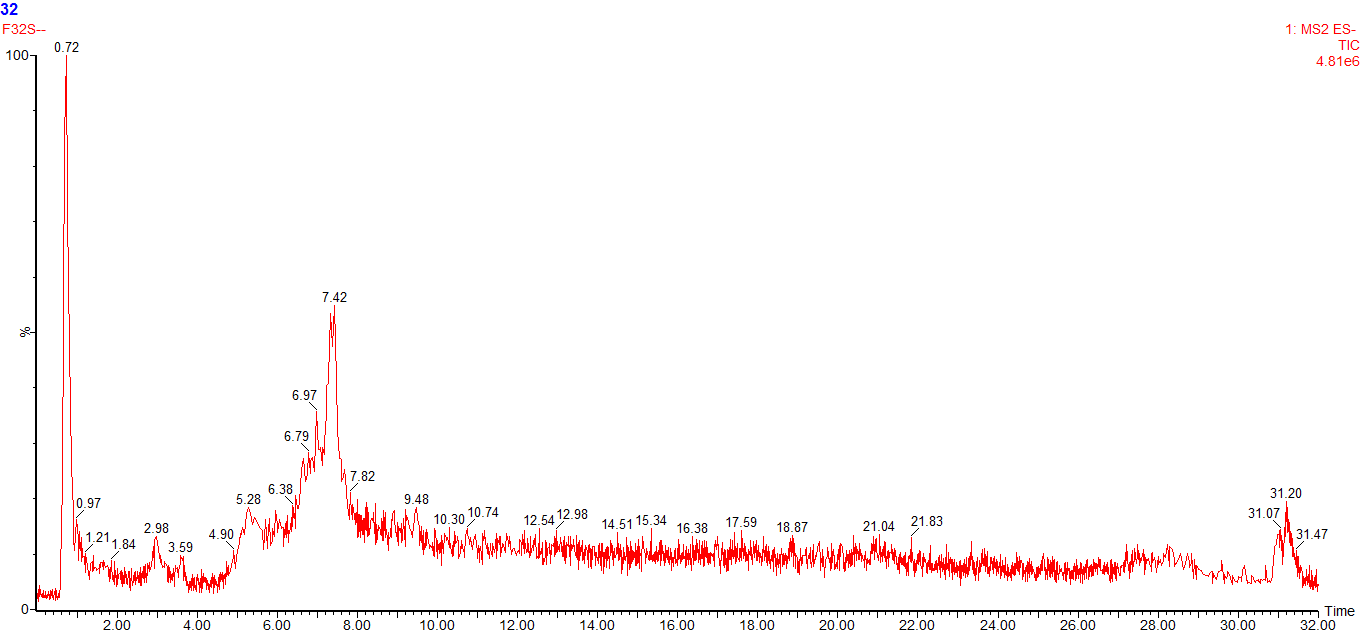** |
| --- |
| **B**  **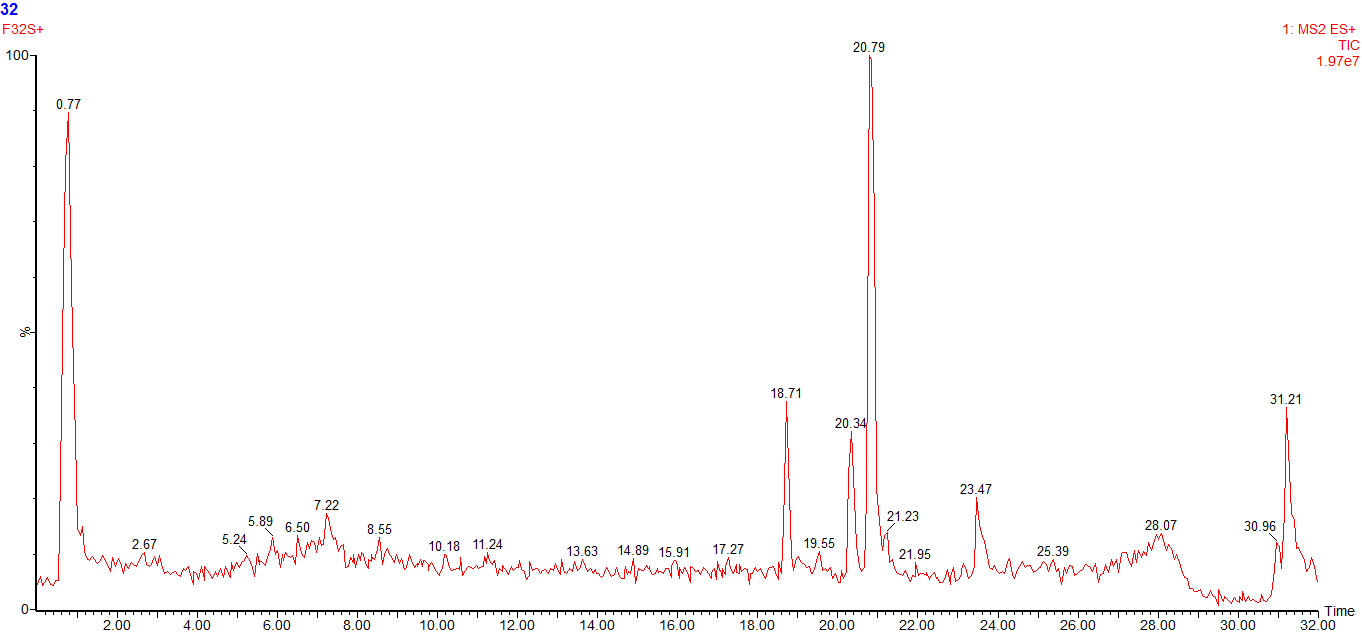** |

**Figure S8. Total ion chromatogram (TIC) of the roots water extract of *S. parviflora* in negative ion mode (A) and positive ion mode (B)**

*Phytochemical analysis by UPLC-ESI-MS/MS Analysis*

The phytochemical profile of *S. parviflora* aerial parts and roots extracts was analyzed using high-performance liquid chromatography coupled with ESI-MS/MS detection, following a previously reported method (Aly et al., 2024a; Aly et al., 2023a; Aly et al., 2023b). This method facilitated tentative identification of phytoconstituents according to their molecular weights. The plant extract (100 *μ*g/mL) was solubilized in HPLC-grade methanol and subsequently filtered through a membrane disc (0.20 *μ*m). Subsequently, the filtrate (10 *μ*L) was put into UPLC-ESI-MS/MS. The utilized HPLC apparatus possesses the subsequent specifications: Waters® equipped with a reversed-phase C-18 column (ACQUITY UPLC-BEH C-18, particle size about 1.7 μm, dimensions = 2.1 × 50 mm). Before injection, the mobile phase was subjected to filtration using a membrane disc filter (0.2 μm) and sonication. The elution duration was 35 minutes, employing gradient elution with water and methanol acidified with 0.1% formic acid at a flow rate of 0.2 mL/min. Positive and negative ions were obtained using ESI-MS on an XEVO TQD triple quadrupole instrument. Full scan MS1 in the range m/z 100–1000 (positive and negative modes separately) to obtain precursor ion masses. For putative identification, we then performed product ion scans (MS/MS) on selected precursor ions that appeared as major or characteristic peaks. These precursor ions were chosen manually based on their relative abundance and expected classes of phytoconstituents. Then, the fragmentation patterns were compared with literature-reported data for the same or similar compounds. The HPLC unit and mass spectrometer were provided by Waters® Corporation, Milford, MA 01757, U.S.A. Edwards®, U.S.A., supplied the vacuum pump at desolvation temperatures of 150 and 440 °C. The mass spectra were acquired utilizing Masslynx 4.1 software within an ESI range of m/z 100–1000. To preliminarily identify the acquired mass spectra, the fragmentation patterns were compared with reported data in the literature.

*Total phenolic and flavonoid content*

The total phenolic content was determined by employing the methods given in the literature with some modification. Sample solution (0.25 mL) was mixed with diluted Folin–Ciocalteu reagent (1 mL, 1:9, v/v) and shaken vigorously. After 3 min, Na2CO3 solution (0.75 mL, 1%) was added and the sample absorbance was read at 760 nm after a 2 h incubation at room temperature. The total phenolic content was expressed as milligrams of gallic acid equivalents (mg GAE/g extract)[1].

The total flavonoid content was determined using the AlCl3 method. Briefly, sample solution (1 mL) was mixed with the same volume of aluminum trichloride (2%) in methanol. Similarly, a blank was prepared by adding sample solution (1 mL) to methanol (1 mL) without AlCl3. The sample and blank absorbances were read at 415 nm after a 10 min incubation at room temperature. The absorbance of the blank was subtracted from that of the sample. Rutin was used as a reference standard and the total flavonoid content was expressed as milligrams of rutin equivalents (mg RE/g extract) [1]

*Determination of Antioxidant and Enzyme Inhibitory Effects*

Antioxidant (DPPH and ABTS radical scavenging, reducing power (CUPRAC and FRAP), phosphomolybdenum and metal chelating (ferrozine method)) and enzyme inhibitory activities (cholinesterase (Elmann’s method), tyrosinase (dopachrome method), α-amylase (iodine/potassium iodide method), α -glucosidase (chromogenic PNPG method) and pancreatic lipase (*p*-nitrophenyl butyrate (p-NPB) method) were determined using the methods previously described by Uysal et al. [1] and Grochowski et al. [2]

For the DPPH (1,1-diphenyl-2-picrylhydrazyl) radical scavenging assay: Sample solution was added to 4 mL of a 0.004% methanol solution of DPPH. The sample absorbance was read at 517 nm after a 30 min incubation at room temperature in the dark. DPPH radical scavenging activity was expressed as milligrams of trolox equivalents (mg TE/g extract).

For ABTS (2,2′-azino-bis(3-ethylbenzothiazoline) 6-sulfonic acid) radical scavenging assay: Briefly, ABTS+ was produced directly by reacting 7 mM ABTS solution with 2.45 mM potassium persulfate and allowing the mixture to stand for 12–16 in the dark at room temperature. Prior to beginning the assay, ABTS solution was diluted with methanol to an absorbance of 0.700 ± 0.02 at 734 nm. Sample solution was added to ABTS solution (2 mL) and mixed. The sample absorbance was read at 734 nm after a 30 min incubation at room temperature. The ABTS radical scavenging activity was expressed as milligrams of Trolox equivalents (mg TE/g extract).

For CUPRAC (cupric ion reducing activity) activity assay: Sample solution was added to premixed reaction mixture containing CuCl2 (1 mL, 10 mM), neocuproine (1 mL, 7.5 mM) and NH4Ac buffer (1 mL, 1 M, pH 7.0). Similarly, a blank was prepared by adding sample solution (0.5 mL) to premixed reaction mixture (3 mL) without CuCl2 . Then, the sample and blank absorbances were read at 450 nm after a 30 min incubation at room temperature. The absorbance of the blank was subtracted from that of the sample. CUPRAC activity was expressed as milligrams of trolox equivalents (mg TE/g extract).

For FRAP (ferric reducing antioxidant power) activity assay: Sample solution was added to premixed FRAP reagent (2 mL) containing acetate buffer (0.3 M, pH 3.6), 2,4,6- tris(2-pyridyl)-S-triazine (TPTZ) (10 mM) in 40 mM HCl and ferric chloride (20 mM) in a ratio of 10:1:1 (v/v/v). Then, the sample absorbance was read at 593 nm after a 30 min incubation at room temperature. FRAP activity was expressed as milligrams of Trolox equivalents (mg TE/g extract).

For phosphomolybdenum method: Sample solution was combined with 3 mL of reagent solution (0.6 M sulfuric acid, 28 mM sodium phosphate and 4 mM ammonium molybdate). The sample absorbance was read at 695 nm after a 90 min incubation at 95 °C. The total antioxidant capacity was expressed as millimoles of trolox equivalents (mmol TE/g extract).

For metal chelating activity assay: Briefly, sample solution was added to FeCl2 solution (0.05 mL, 2 mM). The reaction was initiated by the addition of 5 mM ferrozine (0.2 mL). Similarly, a blank was prepared by adding sample solution (2 mL) to FeCl2 solution (0.05 mL, 2 mM) and water (0.2 mL) without ferrozine. Then, the sample and blank absorbances were read at 562 nm after 10 min incubation at room temperature. The absorbance of the blank was sub-tracted from that of the sample. The metal chelating activity was expressed as milligrams of EDTA (disodium edetate) equivalents (mg EDTAE/g extract).

For Cholinesterase (ChE) inhibitory activity assay: Sample solution (was mixed with DTNB (5,5-dithio-bis(2-nitrobenzoic) acid, Sigma, St. Louis, MO, USA) (125 μL) and AChE (acetylcholines-terase (Electric ell acetylcholinesterase, Type-VI-S, EC 3.1.1.7,Sigma)), or BChE (butyrylcholinesterase (horse serum butyrylcholinesterase, EC 3.1.1.8, Sigma)) solution (25 μL) in Tris–HCl buffer (pH 8.0) in a 96-well microplate and incubated for 15 min at 25 °C. The reaction was then initiated with the addition of acetylthiocholine iodide (ATCI, Sigma) or butyrylthiocholine chloride (BTCl, Sigma) (25 μL). Similarly, a blank was prepared by adding sample solution to all reaction reagents without enzyme (AChE or BChE) solution. The sample and blank absorbances were read at 405 nm after 10 min incubation at 25 °C. The absorbance of the blank was subtracted from that of the sample and the cholinesterase inhibitory activity was expressed as galanthamine equivalents (mgGALAE/g extract).

For Tyrosinase inhibitory activity assay: Sample solution was mixed with tyrosinase solution (40 μL, Sigma) and phosphate buffer (100 μL, pH 6.8) in a 96-well microplate and incubated for 15 min at 25 °C. The reaction was then initiated with the addition of L-DOPA (40 μL, Sigma). Similarly, a blank was prepared by adding sample solution to all reaction reagents without enzyme (tyrosinase) solution. The sample and blank absorbances were read at 492 nm after a 10 min incubation at 25 °C. The absorbance of the blank was subtracted from that of the sample and the tyrosinase inhibitory activity was expressed as kojic acid equivalents (mgKAE/g extract).

For α-amylase inhibitory activity assay: Sample solution was mixed with α- amylase solution (ex-porcine pancreas, EC 3.2.1.1, Sigma) (50 μL) in phosphate buffer (pH 6.9 with 6 mM sodium chloride) in a 96-well microplate and incubated for 10 min at 37 °C. After pre-incubation, the reaction was initiated with the addition of starch solution (50 μL, 0.05%). Similarly, a blank was prepared by adding sample solution to all reaction reagents without enzyme (α-amylase) solution. The reaction mixture was incubated 10 min at 37 °C. The reaction was then stopped with the addition of HCl (25 μL, 1 M). This was followed by addition of the iodine-potassium iodide solution (100 μL). The sample and blank absorbances were read at 630 nm. The absorbance of the blank was subtracted from that of the sample and the α-amylase inhibitory activity was expressed as acarbose equivalents (mmol ACE/g extract).

For α-glucosidase inhibitory activity assay: Sample solution was mixed with glutathione (50 μL), α-glucosidase solution (from Saccharomyces cerevisiae, EC 3.2.1.20, Sigma) (50 μL) in phosphate buffer (pH 6.8) and PNPG (4-N-trophenyl-α-Dglucopyranoside,

Sigma) (50 μL) in a 96-well microplate and incubated for 15 min at 37 °C. Similarly, a blank was prepared by adding sample solution to all reaction reagents without enzyme (α-glucosidase) solution. The reaction was then stopped with the addition of sodium carbonate (50 μL, 0.2 M). The sample and blank absorbances were read at 400 nm. The absorbance of the blank was subtracted from that of the sample and the α-glucosidase inhibitory activity was expressed as acarbose equivalents (mmol ACE/g extract).

**References**

1. Uysal, S.; Zengin, G.; Locatelli, M.; Bahadori, M. B.; Mocan, A.; Bellagamba, G.; De Luca, E.;Mollica, A.; Aktumsek, A., Cytotoxic and enzyme inhibitory potential of two Potentilla species(P. speciosa L. and P. reptans Willd.) and their chemical composition. *Frontiers in pharmacology* **2017,** 8, 290.
2. Grochowski, D. M.; Uysal, S.; Aktumsek, A.; Granica, S.; Zengin, G.; Ceylan, R.; Locatelli, M.;Tomczyk, M., In vitro enzyme inhibitory properties, antioxidant activities, and phytochemicalprofile of Potentilla thuringiaca. *Phytochemistry Letters* **2017,** 20, 365-372.
